# Distributive laws for monotone specifications

**DOI:** 10.1007/s00236-019-00333-x

**Published:** 2019-02-22

**Authors:** Jurriaan Rot

**Affiliations:** Radboud University, Toernooiveld 212, Nijmegen, The Netherlands

## Abstract

Turi and Plotkin introduced an elegant approach to structural operational semantics based on universal coalgebra, parametric in the type of syntax and the type of behaviour. Their framework includes abstract GSOS, a categorical generalisation of the classical GSOS rule format, as well as its categorical dual, coGSOS. Both formats are well behaved, in the sense that each specification has a unique model on which behavioural equivalence is a congruence. Unfortunately, the combination of the two formats does not feature these desirable properties. We show that *monotone* specifications—that disallow negative premises—do induce a canonical distributive law of a monad over a comonad, and therefore a unique, compositional interpretation.

## Introduction

Structural operational semantics (SOS) is an expressive and popular framework for defining the operational semantics of programming languages and calculi. There is a wide variety of specification formats that syntactically restrict the full power of SOS, but guarantee certain desirable properties to hold [1]. A famous example is the so-called GSOS format [5]. Any GSOS specification induces a unique interpretation which is compositional with respect to (strong) bisimilarity.

In their seminal paper [30], Turi and Plotkin introduced an elegant mathematical approach to structural operational semantics, where the type of syntax is modeled by an endofunctor $\varSigma$ and the type of behaviour is modeled by an endofunctor *B*. Operational semantics is then given by a *distributive law* of $\varSigma$ over *B*. In this context, models are *bialgebras*, which consist of a $\varSigma$-algebra and a *B*-coalgebra over a common carrier. One major advantage of this framework over traditional approaches is that it is parametric in the type of behaviour. Indeed, by instantiating the theory to a particular functor *B*, one can obtain well behaved specification formats for probabilistic and stochastic systems, weighted transition systems, streams, and many more [4, 16, 17].

Turi and Plotkin introduced several kinds of natural transformations involving $\varSigma$ and *B*, the most basic one being of the form $\varSigma B \Rightarrow B \varSigma$. If *B* is a functor representing labelled transition systems, then a typical rule that can be represented in this format is the following:

1 $$\begin{aligned} \frac{x \xrightarrow {a} x' \qquad y \xrightarrow {a} y'}{x \otimes y \xrightarrow {a} x' \otimes y'} \end{aligned}$$

This rule should be read as follows: if *x* can make an *a*-transition to $x'$, and *y* an *a*-transition to $y'$, then $x \otimes y$ can make an *a*-transition to $x' \otimes y'$. Any specification of the above kind induces a unique *supported model*, which is a *B*-coalgebra over the initial algebra of $\varSigma$. If $\varSigma$ represents a signature and *B* represents labelled transition systems, then this model is a transition system of which the state space is the set of closed terms in the signature, and, informally, a term makes a transition to another term if and only if there is a rule in the specification justifying this transition.

A more interesting kind is an *abstract GSOS specification*, which is a natural transformation of the form $\varSigma (B \times \mathsf {Id}) \Rightarrow B\varSigma ^*$, where $\varSigma ^*$ is the *free monad* for $\varSigma$ (assuming it exists). If *B* is a functor that models (image-finite) transition systems, and $\varSigma$ is a functor representing a signature, then such specifications correspond to classical GSOS specifications [4, 30]. As opposed to the basic format, GSOS rules allow complex terms in conclusions, as in the following rule specifying a constant *c*:

2 $$\begin{aligned} \frac{}{c \xrightarrow {a} \sigma (c)} \end{aligned}$$

where $\sigma$ is some other operator in the signature (represented by $\varSigma$), which can itself be defined by some GSOS rules. The term $\sigma (c)$ is constructed from a constant and a unary operator from the signature, as opposed to the conclusion $x' \otimes y'$ of the rule in (1), which consists of a single operator and variables. Indeed, the free monad $\varSigma ^*$ occurring in an abstract GSOS specification is precisely what allows a complex term such as $\sigma (c)$ in the conclusion.

Dually, one can consider *coGSOS specifications*, which are of the form $\varSigma B^\infty \Rightarrow B(\varSigma + \mathsf {Id})$, where $B^\infty$ is the cofree comonad for *B* (assuming it exists). In the case of image-finite labelled transition systems, this format corresponds to the *safe ntree format* [30]. A typical coGSOS rule is the following:

3 $$\begin{aligned} \frac{x \xrightarrow {a} x' \qquad x' /\!\!\!\!\!\xrightarrow {a} }{\sigma (x) \xrightarrow {a} x'} \end{aligned}$$

This rule uses two steps of lookahead in the premise; this is supported by the cofree comonad $B^\infty$ in the natural transformation. The symbol $x' /\!\!\!\!\!\xrightarrow {a}$ represents a *negative premise*, which is satisfied whenever $x'$ does not make an *a*-transition.

Both GSOS and coGSOS specifications induce *distributive laws*, and as a consequence they induce unique supported models on which behavioural equivalence is a congruence. The two formats are incomparable in terms of expressive power: GSOS specifications allow rules that involve complex terms in the conclusion, whereas coGSOS allows arbitrary lookahead in the arguments. It is straightforward to *combine* GSOS and coGSOS as a natural transformation of the form $\varSigma B^\infty \Rightarrow B\varSigma ^*$, called a *biGSOS specification*, generalising both formats. However, such specifications are, in some sense, too expressive: they do not induce unique supported models, as already observed in [30]. For example, the rules (2) and (3) above (which are GSOS and coGSOS respectively) can be combined into a single biGSOS specification. Suppose this combined specification has a model. By the axiom for *c*, there is a transition $c \xrightarrow {a} \sigma (c)$ in this model. However, is there a transition $\sigma (c) \xrightarrow {a} \sigma (c)$? If there is not, then by the rule for $\sigma$, there is; but if there is such a transition, then it is not derivable, so it is not in the model! Thus, a supported model does not exist. In fact, it was recently shown that, for biGSOS, it is undecidable whether a (unique) supported model exists [19].

The use of negative premises in the above example (and in [19]) is crucial. In the present paper, we introduce the notion of *monotonicity* of biGSOS specifications, generalising monotone abstract GSOS [9]. In the case that *B* is a functor representing labelled transition systems, this corresponds to the absence of negative premises, but the format does allow lookahead in premises as well as complex terms in conclusions. Monotonicity requires an *order* on the functor *B*—technically, our definition of monotonicity is based on the *similarity* order [12] induced on the final coalgebra.

We show that if there is a pointed DCPO structure on the functor *B*, then any monotone biGSOS specification yields a *least* model as its operational interpretation. Indeed, monotone specifications do not necessarily have a unique model, but it is the least model which makes sense operationally, since this corresponds to the natural notion that every transition has a *finite* proof. Our main result is that if the functor *B* has a DCPO structure, then every monotone specification yields a canonical *distributive law* of the free monad for $\varSigma$ over the cofree comonad for *B*. Its unique model coincides with the least supported model of the specification. As a consequence, behavioural equivalence on this model is a congruence.

However, the conditions of these results are a bit too restrictive: they rule out labelled transition systems, the main example. The problem is that the functors typically used to model transition systems either fail to have a cofree comonad (the powerset functor) or to have a DCPO structure (the finite or countable powerset functor). In the final section, we mitigate this problem using the theory of (countably) presentable categories and accessible functors. This allows us to relax the requirement of DCPO structure only to countable sets, given that the functor *B* is countably accessible (this is weaker than being finitary, a standard condition in the theory of coalgebras) and the syntax consists only of countably many operations each with finite arity. In particular, this applies to labelled transition systems (with countable branching) and certain kinds of weighted transition systems.

*Related work* The current paper is an extended version of a conference paper presented at EXPRESS/SOS 2017 [24]. The main new material is proofs of all results, most of which were missing in the conference version; more backgroud material, in particular on biGSOS rule formats; a result showing that the constructed distributive law extends a given biGSOS specification in the sense of [18].

The idea of studying distributive laws of monads over comonads that are not induced by GSOS or coGSOS specifications has been around for some time (e.g., [4]), but, according to a recent overview paper [17], general bialgebraic formats (other than GSOS or coGSOS) which induce such distributive laws have not been proposed so far. In fact, it is shown by Klin and Nachyła that the general problem of extending biGSOS specifications to distributive laws is undecidable [18, 19]. The current paper shows that one does obtain distributive laws from biGSOS specifications when monotonicity is assumed (negative premises are disallowed). A fundamentally different approach to positive formats with lookahead, not based on the framework of bialgebraic semantics but on labelled transition systems modeled very generally in a topos, was introduced in [29]. It is deeply rooted in labelled transition systems, and hence seems incomparable to our approach based on generic coalgebras for ordered functors. An abstract study of distributive laws of monads over comonads and possible morphisms between them is in [22], but it does not include characterisations in terms of simpler natural transformations.

*Structure of the paper* Section 2 contains preliminaries on (co)algebras, and Sect. 3 recalls (abstract) rule formats based on various kinds of distributive laws. In Sect. 4 the notion of similarity on coalgebras is recalled, which is then used in Sect. 5 to define monotone specifications and prove the existence of least supported models. Section 6 contains our main result: canonical distributive laws for monotone biGSOS specifications. In Sect. 7, this result is extended to countably accessible functors.

## Preliminaries

We recall the necessary definitions on order theory, algebras, coalgebras, and distributive laws of monads over comonads. For an introduction to coalgebra see [14, 27]. All of the definitions and results below and most of the examples can be found in [17], which provides an overview of bialgebraic semantics. Unless mentioned otherwise, all functors considered are endofunctors on the category $\mathsf {Set}$ of sets and functions.

*Notation* By $\mathcal {P}$ we denote the (covariant) power set functor; $\mathcal {P}_c$ is the countable power set functor and $\mathcal {P}_f$ the finite power set functor. Given a relation $R \subseteq X \times Y$, we write $\pi _1 :R \rightarrow X$ and $\pi _2 :R \rightarrow Y$ for its left and right projection, respectively. Given another relation $S \subseteq Y \times Z$ we denote the composition of *R* and *S* by $R \circ S$. We let $R^\mathsf {op}= \{(y,x) \mid (x,y) \in R\}$. For a set *X*, we let $\varDelta _X = \{(x,x) \mid x \in X\}$. The graph of a function $f:X \rightarrow Y$ is $\mathsf {Graph}(f) = \{(x,f(x)) \mid x \in X\}$. The image of a set $S \subseteq X$ under *f* is denoted simply by $f(S) = \{f(x) \mid x \in S\}$, and the inverse image of $V \subseteq Y$ by $f^{-1}(V) = \{x \mid f(x) \in V\}$. The pairing of two functions *f*, *g* with a common domain is denoted by $\langle f, g \rangle$ and the copairing (for functions *f*, *g* with a common codomain) by [*f*, *g*]. The set of functions from *X* to *Y* is denoted by $Y^X$. Any relation $R \subseteq Y \times Y$ can be lifted pointwise to a relation on $Y^X$; in the sequel we will simply denote such a pointwise extension by the relation itself, i.e., for functions $f, g :X \rightarrow Y$ we have $f \, R \, g$ iff $f(x) \, R \, g(x)$ for all $x \in X$, or, equivalently, $(f \times g)(\varDelta _X) \subseteq R$. Composition of functors *F*, *G* (of the appropriate type) is denoted by $F \circ G$ or simply by *FG*, and composition of natural transformations $\alpha , \beta$ by $\alpha \circ \beta$, given by $(\alpha \circ \beta )_X = \alpha _X \circ \beta _X$.

*DCPOs* We recall the basic order-theoretic structures that we will use in fixed point constructions; see, e.g., [7] for details. Let $(P,\le )$ be a poset. Given a subset $S \subseteq P$ we denote the least upper bound of *S* by $\bigvee S$, if it exists. A subset $S \subseteq P$ is called *directed* if *S* is non-empty, and every finite subset of *S* has an upper bound in *S*. A *directed complete partial order (DCPO)* is a poset in which every directed subset has a least upper bound (these are sometimes referred to as CPOs, but we prefer DCPO as it emphasises the directedness aspect). Further, a DCPO is called *pointed* if it has a least element $\bot$. In this paper, every DCPO is assumed to be pointed, and thus will sometimes refer to a pointed DCPO simply as a DCPO. A map $f :P \rightarrow Q$ between pointed DCPOs is called *strict* if $f(\bot ) = \bot$, and *continuous* if, for every directed set $S \subseteq P$, *f*(*S*) is again directed, and $f(\bigvee S) = \bigvee f(S)$. We denote by $\mathsf {DCPO}_\bot$ the category of pointed DCPOs and strict continuous maps.

Every monotone map $f :P \rightarrow P$ on a pointed DCPO has a least fixed point $\mu f$ (see, e.g., [7, Theorem 8.22]). Moreover, this can be computed (e.g., Exercise 8.19 in *loc. cit.*) via the ordinal-indexed chain defined for all $\alpha , \lambda$ with $\lambda$ a limit ordinal by:

$$\begin{aligned} f^0(\bot ) = \bot \,, \qquad f^{\alpha +1}(\bot ) = f(f^\alpha (\bot ))\,, \qquad f^\lambda (\bot ) = \bigvee _{\beta < \lambda } f^\beta (\bot ) \,. \end{aligned}$$

This gives rise to a proof technique: to show that a predicate $\Phi$ holds for all $x \in \mu f$, it suffices to show that, if $x \in \Phi$ then $f(x) \in \Phi$, and if $S \subseteq \Phi$ is a directed set then $\bigvee {S} \in \Phi$.

### Algebras and monads

An *algebra* for a functor $\varSigma :\mathsf {Set}\rightarrow \mathsf {Set}$ consists of a set *X* and a function $f :\varSigma X \rightarrow X$. An *(algebra) homomorphism* from $f :\varSigma X \rightarrow X$ to $g :\varSigma Y \rightarrow Y$ is a function $h :X \rightarrow Y$ such that $h \circ f = g \circ \varSigma h$. The category of algebras and their homomorphisms is denoted by $\mathsf {alg}(\varSigma )$.

A *monad* is a triple $\mathcal {T} = (T, \eta , \mu )$ where $T :\mathsf {Set}\rightarrow \mathsf {Set}$ is a functor and $\eta :\mathsf {Id}\Rightarrow T$ and $\mu :TT \Rightarrow T$ are natural transformations such that $\mu \circ T \eta = \mathsf {id}= \mu \circ \eta T$ and $\mu \circ \mu T = \mu \circ T \mu$. An *(Eilenberg-Moore, or EM)-algebra* for $\mathcal {T}$ is a *T*-algebra $f :TX \rightarrow X$ such that $f \circ \eta _X = \mathsf {id}$ and $f \circ \mu _X = f \circ Tf$. We denote the category of EM-algebras by $\mathsf {Alg}(\mathcal {T})$.

We assume that a *free monad*$(\varSigma ^*, \eta , \mu )$ for $\varSigma$ exists; in the sequel we often refer to this monad by the underlying functor $\varSigma ^*$. This means that there is a natural transformation $\iota :\varSigma \varSigma ^* \Rightarrow \varSigma ^*$ such that for any set *X*, the copairing

$$\begin{aligned}{}[\iota _X, \eta _X] :\varSigma \varSigma ^* X + X \rightarrow \varSigma ^* X \end{aligned}$$

is an initial algebra for the functor $\varSigma + X$. By Lambek’s lemma, $[\iota _X, \eta _X]$ is an isomorphism. Any algebra $f :\varSigma X \rightarrow X$ induces a $\varSigma + X$-algebra $[f, \mathsf {id}]$, and therefore by initiality a $\varSigma ^*$-algebra $f^* :\varSigma ^* X \rightarrow X$, which we call the *inductive extension* of *f*. In particular, the inductive extension of $\iota _X$ is $\mu _X$. This construction preserves algebra homomorphisms: if *h* is a homomorphism from a $\varSigma$-algebra *f* to a $\varSigma$-algebra *g*, then it is also a homomorphism from $f^*$ to $g^*$. It establishes (one side of) an isomorphism of categories $\mathsf {alg}(\varSigma ) \cong \mathsf {Alg}(\varSigma ^*)$, where the other direction (from right to left) is given by precomposing with the natural transformation $\kappa$ in:A *signature* is a functor $\varSigma :\mathbb {N} \rightarrow \mathsf {Set}$, where $\mathbb {N}$ is the discrete category of natural numbers. This models a countable collection of operator names with finite arity: for each *n*, $\varSigma (n)$ is the set of operator names of arity *n*.

Any signature $\varSigma$ gives rise to a polynomial $\mathsf {Set}$ endofunctor which, abusing notation, we also denote by $\varSigma$:

$$\begin{aligned} \varSigma X = \coprod _{n \in \mathbb {N}} \varSigma (n) \times X^n \,. \end{aligned}$$

The free monad $\varSigma ^*$ constructs terms, that is, $\varSigma ^*X$ is given by the grammar

$$\begin{aligned} t {:}{:}{=}\, \sigma (t_1, \ldots , t_n) \mid x \end{aligned}$$

where *x* ranges over *X* and $\sigma$ ranges over the operator names (and *n* is the arity of $\sigma$). In particular, $\varSigma ^*\emptyset$ is the set of closed terms over $\varSigma$.

### Coalgebras and comonads

A *coalgebra* for the functor *B* consists of a set *X* and a function $f :X \rightarrow BX$. A *(coalgebra) homomorphism* from $f :X \rightarrow BX$ to $g :Y \rightarrow BY$ is a function $h :X \rightarrow Y$ such that $Bh \circ f = g \circ h$. The category of *B*-coalgebras and their homomorphisms is denoted by $\mathsf {coalg}(B)$. The associated forgetful functor is denoted by $U :\mathsf {coalg}(B) \rightarrow \mathsf {Set}$. An *F*-coalgebra (*Z*, *z*) is called *final* if it is a final object in $\mathsf {coalg}(B)$, i.e., if there exists a unique homomorphism from every *F*-coalgebra into (*Z*, *z*).

A *comonad* is a triple $\mathcal {D} = (D, \epsilon , \delta )$ consisting of a functor $D :\mathsf {Set}\rightarrow \mathsf {Set}$ and natural transformations $\epsilon :D \Rightarrow \mathsf {Id}$ and $\delta :D \Rightarrow DD$ satisfying axioms dual to the monad axioms. The category of Eilenberg-Moore coalgebras for $\mathcal {D}$, defined dually to EM-algebras, is denoted by $\mathsf {CoAlg}(\mathcal {D})$.

We assume that a *cofree comonad*$(B^\infty , \epsilon , \delta )$ for *B* exists. This means that there is a natural transformation $\theta :B^\infty \Rightarrow BB^\infty$ such that $\theta _X$ is a *cofree coalgebra* on the set *X*, that is, the pairing

$$\begin{aligned} \langle \theta _X,\epsilon _X \rangle :B^\infty X \rightarrow (BB^\infty X) \times X \end{aligned}$$

is a final coalgebra for the functor $B \times X$. Any coalgebra $f :X \rightarrow BX$ induces a $B \times X$-coalgebra $\langle f, \mathsf {id}\rangle$, and therefore by finality a $B^\infty$-coalgebra $f^\infty :X \rightarrow B^\infty X$, which we call the *coinductive extension* of *f*. In particular, the coinductive extension of $\theta _X$ is $\delta _X$. Similar to the case of algebras, this construction is functorial, and establishes an isomorphism $\mathsf {coalg}(B) \cong \mathsf {CoAlg}(B^\infty )$. From right to left, this is given by composing with the natural transformation $\nu$ in:

#### Example 2.1

Consider the $\mathsf {Set}$ functor $BX = A \times X$ for a fixed set *A*. Coalgebras for *B* are called *stream systems*. There exists a final *B*-coalgebra, whose carrier can be presented as the set $A^\omega$ of all streams over *A*, i.e., $A^\omega = \{\sigma \mid \sigma :\omega \rightarrow A \}$ where $\omega$ is the set of natural numbers. For a set *X*, $B^\infty X = (A \times X)^\omega$. Given $f :X \rightarrow A \times X$, its coinductive extension $f^\infty :X \rightarrow B^\infty X$ maps a state $x \in X$ to its infinite unfolding. The final coalgebra of $GX = A \times X + 1$ consists of finite and infinite streams over *A*, that is, elements of $A^* \cup A^\omega$. For a set *X*, $G^\infty X = (A \times X)^\omega \cup (A \times X)^* \times X$.

#### Example 2.2

Labelled transition systems are coalgebras for the functor $(\mathcal {P}-)^A$, where *A* is a fixed set of labels. Image-finite transition systems are coalgebras for the functor $(\mathcal {P}_f-)^A$, and coalgebras for $(\mathcal {P}_c-)^A$ are transition systems which have, for every action $a \in A$ and every state *x*, a countable set of outgoing *a*-transitions from *x*. For an LTS $f :X \rightarrow (\mathcal {P}X)^A$, we sometimes write $x \xrightarrow {a} x'$ if $x' \in f(x)(a)$. A coalgebra homomorphism from an LTS *X* to an LTS *Y* is a map $h :X \rightarrow Y$ such that, for all $x \in X$ and $a \in A$:

- if $x \xrightarrow {a} x'$ then $h(x) \xrightarrow {a} h(x')$, and
- if $h(x) \xrightarrow {a} y'$ then there is $x'$ such that $x \xrightarrow {a} x'$ and $h(x') = y'$.

A final coalgebra for $(\mathcal {P}-)^A$ does not exist (so there is no cofree comonad for it). However, both $(\mathcal {P}_f-)^A$ and $(\mathcal {P}_c-)^A$ have a final coalgebra, consisting of (equivalence classes of) possibly infinite rooted trees, edge-labelled in *A*, modulo strong bisimilarity, where for each label, the set of children is finite respectively countable. The cofree comonad of $(\mathcal {P}_f-)^A$ respectively $(\mathcal {P}_c-)^A$, applied to a set *X*, consist of all trees as above, node-labelled in *X*. The counit $\epsilon _X$ maps such a tree to its root.

#### Example 2.3

A *complete monoid* is a (necessarily commutative) monoid *M* together with an infinitary sum operation consistent with the finite sum [8]. Define the functor $\mathcal {M}:\mathsf {Set}\rightarrow \mathsf {Set}$ by $\mathcal {M}(X) = \{\varphi \mid \varphi :X \rightarrow M\}$ and, for $f :X \rightarrow Y$, $\mathcal {M}(h)(\varphi ) = \lambda y. \sum _{x \in f^{-1}(y)} \varphi (x)$. A *weighted transition system* over a set of labels *A* is a coalgebra $f :X \rightarrow (\mathcal {M}X)^A$. Similar to the case of labelled transition systems, we obtain weighted transition systems whose branching is countable for each label as coalgebras for the functor $(\mathcal {M}_c -)^A$, where $\mathcal {M}_c$ is defined by $\mathcal {M}_c(X) = \{\varphi :X \rightarrow M \mid \varphi (x) \ne 0 \text { for countably many }x \in X\}$. We note that this only requires a countable sum on *M* to be well-defined and, by further restricting to finite support, weighted transition systems are defined for any commutative monoid (see, e.g., [16]).

Labelled transition systems are retrieved by taking the monoid with two elements and logical disjunction as sum. Another example arises by taking the monoid $M = \mathbb {R}^+ \cup \{\infty \}$ of non-negative reals extended with a top element $\infty$, with the supremum operation.

### Distributive laws

In Sect. 3, we will use distributive laws as a common generalisation of several abstract rule formats. For now, we only recall a few basic definitions and properties.

A *distributive law* of a monad $\mathcal {T} = (T, \eta , \mu )$ over a comonad $\mathcal {D} = (D, \epsilon , \delta )$ is a natural transformation $\lambda :TD \Rightarrow DT$ making the following diagrams commute.The axioms ensure compatibility with the monad and comonad structure. For more details of their use in SOS, see [17].

A *lifting* of a functor $T :\mathsf {Set}\rightarrow \mathsf {Set}$ to $\mathsf {CoAlg}(\mathcal {D})$ is a functor $\overline{T}$ making the following commute:where the vertical arrows are the forgetful functor. Further, a monad $(\overline{T}, \overline{\eta }, \overline{\mu })$ on $\mathsf {CoAlg}(\mathcal {D})$ is a lifting of a monad $\mathcal {T} = (T,\eta ,\mu )$ on $\mathsf {Set}$ if

- $\overline{T}$ is a lifting of *T*;
- $$U\overline{\eta } = \eta U$$ and $$U \overline{\mu }= \mu U$$.

A lifting of $\mathcal {T}$ to $\mathsf {coalg}(B)$ is defined similarly. Distributive laws of $\mathcal {T}$ over $\mathcal {D}$ are in one-to-one correspondence with liftings of $(T, \eta , \mu )$ to $\mathsf {CoAlg}(\mathcal {D})$ [15, 30]. If $\mathcal {D}$ is the cofree comonad for *B*, then $\mathsf {CoAlg}(\mathcal {D}) \cong \mathsf {coalg}(B)$, hence a further equivalent condition is that $\mathcal {T}$ lifts to $\mathsf {coalg}(B)$.

Given a distributive law $\lambda :TD \Rightarrow DT$, a $\lambda$-*bialgebra* is a triple (*X*, *a*, *f*) where *X* is a set, *a* is an EM-algebra for $\mathcal {T}$ and *f* is an EM-coalgebra for $\mathcal {D}$, such that the following diagram commutes:Any distributive law $\lambda$ induces, by initiality, a unique coalgebra $h :T\emptyset \rightarrow DT\emptyset$ such that $(T\emptyset , \mu _\emptyset , h)$ is $\lambda$-bialgebra, i.e., making the diagram on the left below commute:Similarly, there is a unique algebra structure $k :TD1 \rightarrow D1$ (where 1 is a singleton, so that *D*1 is the carrier of a final coalgebra) making the diagram on the right commute.

The map *h* is a coalgebra for the comonad *D*. If *D* is the cofree comonad $B^\infty$ of a functor *B*, then *h* corresponds to a *B*-coalgebra; we refer to the latter as the *operational model* of $\lambda$.

The coalgebra $h :T\emptyset \rightarrow DT\emptyset$ plays a special role. As we will see in the next section, in the context of certain rule formats on, e.g., labelled transition systems, it is the labelled transition system over closed terms induced by a given specification. In this context, the semantics arises by finality, as the unique coalgebra morphism $s :T\emptyset \rightarrow D1$ in the diagram on the left:Dually, the algebra *k* is the interpretation of the operations defined in the specification on the final coalgebra. It is easy to show that the same map *s* (defined as the unique map to a final coalgebra) is also an algebra morphism from the initial algebra (as on the right above). This means that behavioural equivalence on *h*, i.e., identification of elements by the ‘semantics map’ *s*, is a congruence (since *s* is an algebra morphism). For a more extensive explanation, see [17, 30].

## Abstract rule formats: GSOS, coGSOS and biGSOS

In this section we recall the abstract rule formats originally introduced in [30], and their combination, called biGSOS in [18].

### GSOS

Probably the most important instance of these formats is *abstract GSOS*, which generalises the classical GSOS format for labelled transition systems [5]. An abstract GSOS specification [30] is a natural transformation of the form

$$\begin{aligned} \rho :\varSigma (B \times \mathsf {Id}) \Rightarrow B\varSigma ^* \, . \end{aligned}$$

This notion gives rise to concrete rule formats for different types of coalgebras, by varying the functor *B*. We recall this for streams and labelled transition systems.

#### Example 3.1

Given a signature $\varSigma$ (Sect. 2.1), a *stream GSOS* rule [11, 17] for an operator $\sigma$ of arity *n* is of the form

$$\begin{aligned} \frac{x_1 \xrightarrow {a_1} x_1' \qquad \ldots \qquad x_n \xrightarrow {a_n} x_n'}{\sigma (x_1, \ldots x_n) \xrightarrow {b} t} \end{aligned}$$

where $x_1, \ldots , x_n, x_1', \ldots , x_1'$ are pairwise distinct variables; *t* is a term over these variables and the signature $\varSigma$; and $a_1, \ldots , a_n, b \in \mathbb {R}$.

A stream GSOS specification is a collection of stream GSOS rules over a common signature such that, for every operator $\sigma$ (of arity *n*) and every sequence $(a_1, \ldots , a_n) \in \mathbb {R}^n$, there is exactly one rule with premises $x_1 \xrightarrow {a_1} x_1'$, ..., $x_n \xrightarrow {a_n} x_n'$.

Let $BX = \mathbb {R}\times X$, and let $\varSigma$ be a signature. Every stream GSOS specification gives rise to an abstract GSOS specification $\varSigma (B \times \mathsf {Id}) \Rightarrow B \varSigma ^*$ involving these functors, and conversely, every abstract GSOS specification arises in this way [17]. Rather than recalling the exact construction, we consider an example of basic operations on streams: a binary sum $\oplus$, (shuffle) product $\otimes$ and a constant ${\overline{r}}$ for each $r \in \mathbb {R}$. The following stream GSOS rules define their semantics (corresponding to the usual behavioural differential equations [26]):

$$\begin{aligned} \frac{x \xrightarrow {r} x' \qquad y \xrightarrow {s} y'}{x \oplus y \xrightarrow {r + s} x' \oplus y'} \qquad \frac{x \xrightarrow {r} x' \qquad y \xrightarrow {s} y'}{x \otimes y \xrightarrow {r \times s} (x' \otimes y) \oplus (x \otimes y')} \qquad \frac{}{{\overline{r}} \xrightarrow {r} {\overline{0}}} \end{aligned}$$

To model this as an abstract GSOS specification, we take $\varSigma X = (X \times X) + (X \times X) + \mathbb {R}$, and denote the left coproduct injection of a pair (*x*, *y*) by $x \oplus y$, the middle by $x \otimes y$ and the right (for an element $r \in \mathbb {R}$) by ${\overline{r}}$. Then the natural transformation $\rho :\varSigma (B \times \mathsf {Id}) \Rightarrow B\varSigma ^*$ (with $BX = \mathbb {R}\times X$) corresponding to the above specification is given on a component *X* by

$$\begin{aligned} \rho _X((x,r,x') \oplus (y,s,y'))&= (r + s, (x' \oplus y')) \\ \rho _X((x,r,x') \otimes (y,s,y'))&= (r \times s, (x' \otimes y) \oplus (x \otimes y')) \\ \rho _X({\overline{r}})&= (r, {\overline{0}}) \end{aligned}$$

#### Example 3.2

Given a signature $\varSigma$ , a *GSOS* rule [5] for an operator $\sigma$ of arity *n* is of the form

4 $$\begin{aligned} \frac{\{x_{i_j} {\mathop {\rightarrow }\limits ^{a_j}} y_j\}_{j = 1..m} \qquad \{x_{i_k} /\!\!\!\!\!\xrightarrow {b_{k}}\}_{k = 1..l}}{\sigma (x_1, \ldots , x_n) {\mathop {\rightarrow }\limits ^{c}} t} \end{aligned}$$

where *m* and *l* are the number of positive and negative premises respectively; $a_1, \ldots , a_m, b_1, \ldots , b_l, c \in A$ are labels; $x_1, \ldots , x_n$, $y_1, \ldots , y_m$ are pairwise distinct variables, and *t* is a term over these variables and the signature $\varSigma$.

A GSOS *specification* is a collection of rules satisfying an image-finiteness condition. As first observed in [30], and proved in detail in [4], specifications in the GSOS format are generalised by abstract GSOS specifications, where $\varSigma$ models the signature and $BX = (\mathcal {P}_fX)^A$.

A *model* of an abstract GSOS specification $\rho :\varSigma (B \times \mathsf {Id}) \Rightarrow B \varSigma ^*$ is a triple (*X*, *a*, *f*) where *X* is a set, $a :\varSigma X \rightarrow X$ an algebra and $f :X \rightarrow BX$ a coalgebra, such that the diagram on the left below commutes:Of particular interest are models on the initial algebra, i.e., where the algebra part is given by $\iota _\emptyset :\varSigma \varSigma ^* \emptyset \rightarrow \varSigma ^* \emptyset$. It turns out that, in this case, there is a *unique* coalgebra structure *f* turning $(\varSigma ^* \emptyset , \iota _\emptyset , f)$ into a model, as depicted on the right above. We call this the *supported model* of the abstract GSOS specification $\rho$, since it generalises the usual notion of supported model for labelled transition systems, informally stating that a transition $\sigma (t_1, \ldots , t_n) \xrightarrow {a} t'$ is in the model iff it is provable by the rules.

The uniqueness of the supported model follows from the theory of distributive laws, using that every abstract GSOS specification $\rho$ induces a distributive law $\lambda :\varSigma ^* B^\infty \Rightarrow B^\infty \varSigma ^*$ of the free monad over the cofree comonad [17, 20].

It also follows from this correspondence that behavioural equivalence on the supported model of $\rho$ is a congruence.

### coGSOS

The ‘dual’ of GSOS is coGSOS, introduced in [30]. It is used a lot less than GSOS, since it appears most examples (such as the standard operators of the stream calculus [26]) fit in the GSOS format and not in the coGSOS format. A *coGSOS specification* is a natural transformation of the form

$$\begin{aligned} \rho :\varSigma B^\infty \Rightarrow B(\varSigma + \mathsf {Id}) \, . \end{aligned}$$

In case of labelled transition systems, these natural transformations can be obtained from specifications in the *safe ntree* format, which we will see as a special case of a more general situation below (Example 3.6).

#### Example 3.3

In case of streams (i.e., $BX = \mathbb {R}\times X$), coGSOS specifications can be presented concretely as a rule format which allows lookahead in premises, but where the term *t*, the right-hand side of the conclusion, can only be a single operation, or a variable.

Concretely, a *stream coGSOS* rule [17] for an operator $\sigma$ of arity *n* is of the form

$$\begin{aligned} \frac{\{x_1^i \xrightarrow {a_1^i} x_1^{i+1}\}_{i \in \mathbb {N}_{>0}} \qquad \ldots \qquad \{x_n^i \xrightarrow {a_n^i} x_n^{i+1}\}_{i \in \mathbb {N}_{>0}}}{\sigma (x_1, \ldots x_n) \xrightarrow {b} t} \end{aligned}$$

where $\mathbb {N}_{>0}$ is the set of positive natural numbers, $x_1^1, \ldots , x_n^1, x_1^2, \ldots , x_1^2, \ldots$ are pairwise distinct variables; *t* is either a single operator over these variables or a single variable drawn from this set; and $b,a_1^1, \ldots , a_n^1, a_1^2, \ldots , a_n^2, \ldots \in \mathbb {R}$.

Note that coGSOS specifications allow infinitely many premises; in particular, one can inspect the entire argument stream in the premise. This is operationally rather questionable, but for practicular purposes, one may give rules which only mention a finite number of premises for each argument. For instance, consider the following rule:

$$\begin{aligned} \frac{x \xrightarrow {a} x' \qquad x' \xrightarrow {a'} x''}{\mathsf {even}(x) \xrightarrow {a} \mathsf {even}(x'')} \end{aligned}$$

It is straightforward to extend this to a collection of rules in the coGSOS format, by extending the premises to infinite sequences in all possible ways; see [17] for details.

The notion of (supported) model of a coGSOS specification is defined similarly (dually) to that of a GSOS specification; we will see a more definition that generalises both in the context of the biGSOS format, introduced next. Just like the GSOS case, coGSOS specifications have unique supported models, on which behavioural equivalence is a congruence [17].

### biGSOS

The abstract GSOS and coGSOS formats have a straightforward common generalisation, called *biGSOS* in [18]. This notion, as well as the associated notion of model which we define later, subsumes both abstract GSOS and coGSOS.

#### Definition 3.4

A *biGSOS specification* is a natural transformation of the form

$$\begin{aligned} \rho :\varSigma B^\infty \Rightarrow B \varSigma ^* \,. \end{aligned}$$

#### Example 3.5

For streams (i.e., $BX = \mathbb {R}\times X$), a concrete presentation of the biGSOS format is very similar to the (coGSOS) format given in Example 3.3; however, the term *t* on the right-hand side of the conclusion of a rule is now allowed to be an arbitrary term over the variables introduced in the premises, rather than just a single operator or variable.

Of course, all GSOS and coGSOS rules form examples of biGSOS rules. As pointed out in [4], a nice example that fits in the biGSOS format but not in one of the other formats, is the $\varDelta _c$ operator from [28], called Laplace–Carson transform (on the left below):

$$\begin{aligned} \frac{x \xrightarrow {r} x' \quad x' \xrightarrow {r'} x''}{\varDelta _c(x) \xrightarrow {r} \varDelta _c(x' \oplus (X \otimes x''))} \qquad \frac{}{X \xrightarrow {0} {\overline{1}}} \end{aligned}$$

It makes use of the shuffle product, sum (Example 3.1) and the operator *X* (defined above, using the constant ${\overline{1}}$), so together with those operations it forms a biGSOS specification.

#### Example 3.6

If $BX = (\mathcal {P}_fX)^A$, then one can obtain biGSOS specifications from concrete rules in the *ntree* format. An *ntree* rule (as taken from [17]) for an operator $\sigma$ with arity *n* is of the form

5 $$\begin{aligned} \frac{\{z_i {\mathop {\rightarrow }\limits ^{a_i}} y_i\}_{i \in I} \qquad \{w_j /\!\!\!\!\!\xrightarrow {b_{j}}\}_{j \in J}}{\sigma (x_1, \ldots , x_n) {\mathop {\rightarrow }\limits ^{c}} t} \end{aligned}$$

where *I* and *J* are countable possibly infinite sets, the $z_i$, $y_i$, $w_j$, $x_k$ are variables, and $b_j, c, a_i \in A$; the $x_k$ and $y_i$ are all distinct and they are the only variables that occur in the rule; the dependency graph of premise variables (where positive premises are seen as directed edges) is well-founded, and *t* is a term over the variables. An ntree rule is called *safe* if *t* is either a single variable or a term built of a single operator from the signature and the variables.

As stated in [30], every ntree specification (a collection of ntree rules with a certain image-finiteness condition) induces a biGSOS specification where $\varSigma$ models the signature and $BX = (\mathcal {P}_fX)^A$. Safe ntree rules induce coGSOS specifications. The construction of a biGSOS specification $\rho$ from an ntree specification is sketched in [30]. Here, we fill in some of the details. Note that it is not known whether there is a converse, i.e., whether every biGSOS specification is derived from an ntree specification [17].

First, note that by the conditions imposed on ntree rules (5), the premises give rise to collection of *n* disjoint trees with roots $x_1, \ldots , x_n$ respectively, edge-labelled in *A* and node-labelled in variables, with a unique label for each node. Let *V* be the set of variables that occur in the nodes of these trees. Given $b \in B^\infty X$ and a map $h :V \rightarrow B^\infty X$, we write $x_i \models _h b_i$ if

1. $$h(x_i) = b_i$$, and
2. if $x \xrightarrow {a} x'$ (in the rooted tree induced by the premises) then $h(x) \xrightarrow {a} h(x')$, and
3. if $x /\!\!\!\!\!\xrightarrow {a}$ (an explicit negative premise in the rule) then $h(x) /\!\!\!\!\!\xrightarrow {a}$ (no *a*-transition from *h*(*x*)).

Now, $\rho :\varSigma B^\infty \Rightarrow (\mathcal {P}_f\varSigma ^* X)^A$ (for $BX = (\mathcal {P}_fX)^A$) is defined as follows: $u \in \rho _X(\sigma (b_1, \ldots , b_n))(a)$ iff $u = \varSigma ^*(\epsilon _X \circ h)(t)$ for some $t \in \varSigma ^*X$ and *h* as above such that there is a rule of the form (5) with for each *i*, $x_i \models _h b_i$. We prove naturality in Appendix A.

A triple (*X*, *a*, *f*) consisting of a set *X*, an algebra $a :\varSigma X \rightarrow X$ and a coalgebra $f :X \rightarrow BX$ (i.e., a bialgebra) is called a $\rho$-*model* if the diagram on the left below commutes.6In particular, we define a *supported* model as a $\rho$-model on the initial algebra, i.e., a coalgebra $f :\varSigma ^* \emptyset \rightarrow B\varSigma ^* \emptyset$ such that the diagram on the right above commutes.

Contrary to the case of GSOS and coGSOS, in general there is no (unique) supported model. This is witnessed, for instance, by the example specification in the introduction. In Example 3.7, we shall see that biGSOS specifications may also have multiple supported models, and that behavioural equivalence is not even a congruence, in general.

#### Example 3.7

In this example we consider a signature with constants *c* and *d*, and unary operators $\sigma$ and $\tau$. Consider the specification (represented by concrete rules) on labelled transition systems where *c* and *d* are not assigned any behaviour, and $\sigma$ and $\tau$ are given by the following rules:

$$\begin{aligned} \frac{x \xrightarrow {a} x' \qquad x' \xrightarrow {a} x''}{\sigma (x) \xrightarrow {a} x''} \qquad \frac{}{\tau (x) \xrightarrow {a} \sigma (\tau (x))} \end{aligned}$$

The behaviour of $\tau (x)$ is independent of its argument *x*. Which transitions can occur in a supported model? First, for any *t* there is a transition $\tau (t) \xrightarrow {a} \sigma (\tau (t))$. Moreover, a transition $\sigma (\tau (t)) \xrightarrow {a} t''$*can* be in the model, although it does not need to be. But if it is there, it is supported by an *infinite* proof.

In fact, one can easily construct a model in which the behaviour of $\sigma (\tau (c))$ is different from that of $\sigma (\tau (d))$—for example, a model where $\sigma (\tau (c))$ does not make any transitions, whereas $\sigma (\tau (d)) \xrightarrow {a} t$ for some *t*. Then behavioural equivalence is not a congruence; *c* is bisimilar to *d*, but $\sigma (\tau (c))$ is not bisimilar to $\sigma (\tau (d))$.

The above example features a specification that has many different interpretations as a supported model, on the set of closed terms. We are interested in the *least* model, since that only features *finite* proofs. It is sensible to speak about the least model of this specification, since it does not contain any negative premises. More generally, absence of negative premises can be defined based on an ordered functor and the induced similarity order, as we will see in the next sections.

Every GSOS and coGSOS specification induces a distributive law. Using this, one can prove that such specifications have unique supported models on which behavioural equivalence is a congruence. The above examples of biGSOS specifications, where some do not have a model and some have multiple models, suggest that the correspondence with distributive laws does not go through for biGSOS specifications. To make this slightly more precise, we say a distributive law $\lambda :\varSigma ^* B^\infty \Rightarrow B^\infty \varSigma ^*$ of monad over comonad *extends* a biGSOS specification $\rho :\varSigma B^\infty \Rightarrow B \varSigma ^*$ if the following diagram commutes [18]:Klin and Nachyła [18] show that, in case of stream systems or labelled transition systems, it is undecidable whether a given biGSOS specification $\rho$ extends (uniquely) to a distributive law $\lambda$. In fact, not even the full power of biGSOS is used there: every individual rule is required to be either in the GSOS or in the coGSOS format.

The current paper takes a different approach, by disallowing negative premises—which are crucial in the proof of Klin and Nachyła. Further, we will construct a canonical distributive law that extends a given biGSOS specification (satisfying a monotonicity condition generalising absence of negative premises), but we make no claims about uniqueness.

## Similarity on cofree coalgebras

In this section, we recall the notion of *simulation* of coalgebras from [12], and prove a few basic results concerning the similarity preorder on final coalgebras. This similarity preorder will be used in Sect. 5 to formulate what it means for a biGSOS specification to be monotone.

Throughout this section, let $B :\mathsf {Set}\rightarrow \mathsf {Set}$ be a functor for which a cofree coalgebra $B^\infty$ exists. The (canonical) *relation lifting* of *B* is defined on a relation $R \subseteq X \times Y$ by

$$\begin{aligned} \mathsf {Rel}(B)(R) = \{(b,c) \in BX \times BY \mid \exists d \in BR.\, B\pi _1(d) = b \text { and } B\pi _2(d) = c \} \,. \end{aligned}$$

It thus extends a relation $R \subseteq X \times Y$ to a relation $\mathsf {Rel}(B)(R) \subseteq BX \times BY$, by taking the image of *BR* along the pairing $\langle B\pi _1, B\pi _2 \rangle :BR \rightarrow BX \times BY$. For instance, for the functor $B(X) = A \times X$, we have

$$\begin{aligned} \mathsf {Rel}(B)(R) = \{((a,x),(a,y)) \mid a \in A \text { and } (x,y) \in R\} \, . \end{aligned}$$

Relation lifting can be used to define a notion of bisimulation, which we recall, as the notion of simulation that we use later is a variation on it. Let (*X*, *f*) and (*Y*, *g*) be *B*-coalgebras. A relation $R \subseteq X \times X$ is a *bisimulation* if $R \subseteq (f \times g)^{-1}(\mathsf {Rel}(B)(R))$. For a detailed account of bisimulations and relation lifting, see, e.g., [13]. We recall the following standard properties, which will be useful later.

### Lemma 4.1

For any functor $F :\mathsf {Set}\rightarrow \mathsf {Set}$ and any $R \subseteq X \times X'$, $S \subseteq Y \times Y'$:

1. $$\mathsf {Rel}(F)(\varDelta _X) = \varDelta _{FX}$$.
2. For any $R' \subseteq X \times X'$: if $R \subseteq R'$ then $\mathsf {Rel}(F)(R) \subseteq \mathsf {Rel}(F)(R')$.
3. For any $$f :X \rightarrow Y$$, $$g :X' \rightarrow Y'$$: $$(F f \times F g) (\mathsf {Rel}(F)(R)) \subseteq \mathsf {Rel}(F)((f \times g)(R))$$.
4. $$\mathsf {Rel}(F)(R)^\mathsf {op}= \mathsf {Rel}(F)(R^\mathsf {op})$$.
5. If *F* preserves weak pullbacks, then for any $f :X \rightarrow Y$: $\mathsf {Rel}(F)(\mathsf {Graph}(f)) = \mathsf {Graph}(Ff)$ .
6. For any $f :X \rightarrow Y$, $g :X' \rightarrow Y'$: if $f \mathrel {S} g$ then $F(f) \mathrel {\mathsf {Rel}(F)(S)} F(g)$,

### Proof

The first five properties are standard (see, e.g., [13]). For (6), by assumption we have $(f \times g)(\varDelta _X) \subseteq {S}$. By (2) this implies $\mathsf {Rel}(F)((f \times g)(\varDelta _X)) \subseteq \mathsf {Rel}(F)(S)$. Further, by (1) and (3), we have $(F f \times F g)(\varDelta _{F X}) = (F f \times F g)(\mathsf {Rel}(F)(\varDelta _X)) \subseteq \mathsf {Rel}(F)((f \times g)(\varDelta _X))$. Thus $(F f \times F g)(\varDelta _{F X}) \subseteq \mathsf {Rel}(F)(S)$ as desired. $\square$

An *ordered functor* is a pair $(B, \sqsubseteq )$ of functors $B :\mathsf {Set}\rightarrow \mathsf {Set}$ and $\sqsubseteq :\mathsf {Set}\rightarrow \mathsf {PreOrd}$ such thatcommutes, where the arrow from $\mathsf {PreOrd}$ to $\mathsf {Set}$ is the forgetful functor mapping a preorder to its carrier. Concretely, this means:

- For any set *X*, there is a preorder ${\sqsubseteq _{BX}} \subseteq BX \times BX$;
- For any map $f :X \rightarrow Y$, *Bf* is monotone, i.e., $b \sqsubseteq _{BX} c$ implies $Bf(b) \sqsubseteq _{BY} Bf(c)$.

Let $(B, \sqsubseteq )$ be an ordered functor. The *lax relation lifting*$\mathsf {Rel}_{\sqsubseteq }$ is defined as follows:

$$\begin{aligned} \mathsf {Rel}_{\sqsubseteq }(B)(R \subseteq X \times Y) = {\sqsubseteq _{BX}} \circ \mathsf {Rel}(B)(R) \circ {\sqsubseteq _{BY}} \, . \end{aligned}$$

Let (*X*, *f*) and (*Y*, *g*) be *B*-coalgebras. A relation $R \subseteq X \times Y$ is a *simulation* (between *f* and *g*) if $R \subseteq (f \times g)^{-1}(\mathsf {Rel}_{\sqsubseteq }(B)(R))$. The greatest simulation between coalgebras *f* and *g* is called *similarity*, denoted by $\lesssim _f^g$, or $\lesssim _f$ if $f=g$, or simply $\lesssim$ if *f* and *g* are clear from the context.

Given a set *X* and an ordered functor $(B, \sqsubseteq )$, we define the ordered functor $(B \times X, {\widetilde{\sqsubseteq }})$ by

$$\begin{aligned} (b,x) \mathrel {{\widetilde{\sqsubseteq }}_{BX}} (c,y) \quad \text { iff } \quad b \sqsubseteq _{BX} c \text { and }x=y \,. \end{aligned}$$

The induced notion of simulation can naturally be expressed in terms of the original one:

### Lemma 4.2

Let $\lesssim$ be the similarity relation between coalgebras $\langle f, f' \rangle :X \rightarrow BX \times Z$ and $\langle g, g' \rangle :X \rightarrow BX \times Z$. Then for any relation $R \subseteq X \times X$:

$$\begin{aligned} R \subseteq (\langle f, f'\rangle \times \langle g, g' \rangle )^{-1}(\mathsf {Rel}_{{\widetilde{\sqsubseteq }}}(B \times Z)(R)) \end{aligned}$$

iff $R \subseteq (f\times g)^{-1}(\mathsf {Rel}_{\sqsubseteq }(B)(R))$ and for all $(x,y) \in R$: $f'(x) = g'(x)$.

### Proof

We compute a more concrete presentation of $\mathsf {Rel}_{{\widetilde{\sqsubseteq }}}(B \times Z)(R)$:

$$\begin{aligned} \mathsf {Rel}_{{\widetilde{\sqsubseteq }}}(B \times Z)(R)&= {\widetilde{\sqsubseteq }} \circ \mathsf {Rel}(B \times Z)(R) \circ {\widetilde{\sqsubseteq }} \\&= {\widetilde{\sqsubseteq }} \circ \{((B \times Z)(\pi _1)(z), (B \times Z)(\pi _2)(z)) \mid z \in (B \times Z)(R) \} \circ {\widetilde{\sqsubseteq }} \\&= {\widetilde{\sqsubseteq }} \circ \{((B\pi _1(u),v), (B\pi _2(u),v)) \mid u \in BR ,\, v \in Z \} \circ {\widetilde{\sqsubseteq }} \\&= {\widetilde{\sqsubseteq }} \circ \{((b,v), (c,v)) \mid (b,c) \in \mathsf {Rel}(B)(R) ,\, v \in Z \} \circ {\widetilde{\sqsubseteq }} \\&= \{((b,v),(c,v)) \mid (b,c) \in \mathsf {Rel}_{\sqsubseteq }(B)(R),\, v \in Z\} \,. \end{aligned}$$

The desired equivalence follows easily from this presentation. $\square$

Given an ordered functor $(B, \sqsubseteq )$ we write

$$\begin{aligned} \lesssim _{B^\infty X} \end{aligned}$$

for the similarity order induced by the ordered functor $(B \times X, {\widetilde{\sqsubseteq }})$ on the cofree coalgebra $(B^\infty X, \langle \theta _X, \epsilon _X \rangle )$. We discuss a few examples of ordered functors and similarity; see [12] for many more.

### Example 4.3

For the functor $L_fX = (\mathcal {P}_fX)^A$ ordered by (pointwise) subset inclusion, a simulation as defined above is a (strong) simulation in the standard sense. For elements $p,q \in L_f^\infty X$, we have $p \lesssim _{L_f^\infty X} q$ iff there exists a (strong) simulation *R* between the underlying trees of *p* and *q*, so that pairs in *R* agree on labels in the set *X*. Concretely, for all $p,q \in R$:

- $$\epsilon _X(p) = \epsilon _X(q)$$, and
- if $p \xrightarrow {a} p'$ then there is $q'$ such that $q \xrightarrow {a} q'$ and $(p',q') \in R$.

### Example 4.4

For any $G :\mathsf {Set}\rightarrow \mathsf {Set}$, the functor $B = G + 1$, where $1 = \{\bot \}$, can be ordered as follows: $x \le y$ iff $x = \bot$ or $x=y$, for all $x,y \in BX$. If $G = A \times \mathsf {Id}$ then $B^\infty X$ consists of finite and infinite sequences of the form $x_0 \xrightarrow {a_0} x_1 \xrightarrow {a_1} x_2 \xrightarrow {a_2} \ldots$ with $x_i \in X$ and $a_i \in A$ for each *i* (cf. Example 2.1). For $\sigma ,\tau \in B^\infty X$ we have $\sigma \lesssim _{B^\infty X} \tau$ if $\tau$ does not terminate before $\sigma$ does, and $\sigma$ and $\tau$ agree on labels in *X* and *A* on each position where $\sigma$ is defined.

In the remainder of this section we state a few technical properties concerning similarity on cofree comonads, which will be necessary in the following sections.

### Lemma 4.5

Coalgebra homomorphisms *h*, *k* preserve similarity: if $x \lesssim y$ then $h(x) \lesssim k(y)$.

### Proof

Let $h :X \rightarrow X'$ be a coalgebra homomorphism from (*X*, *f*) to $(X',f')$, and $k :Y \rightarrow Y'$ a coalgebra homomorphism from (*Y*, *g*) to $(Y',g')$. In [12], it is shown that $x \lesssim y$ iff $h(x) \lesssim k(y)$ under the assumption of a stable order (meaning that $\mathsf {Rel}_{\sqsubseteq }(B)$ is a fibred functor); but this is not necessary for the implication from left to right. In fact, the desired implication follows from the abstract theory of bifibrations, see, e.g., [23, Proposition 3.3.7].

For a more concrete proof, we show that the direct image of a simulation under $(h \times k)$ is again a simulation. To this end, let $R \subseteq (f \times g)^{-1}(\mathsf {Rel}_{\sqsubseteq }(B)(R))$. Then

$$\begin{aligned} (h \times k)(R) \subseteq (h \times k)((f \times g)^{-1}(\mathsf {Rel}_{\sqsubseteq }(B)(R))) \end{aligned}$$

and the result follows (i.e., $(h\times k)(R)$ is a simulation) once we show that

$$\begin{aligned} (h \times k)((f \times g)^{-1}(\mathsf {Rel}_{\sqsubseteq }(B)(R))) \subseteq (f' \times g')^{-1}( \mathsf {Rel}_{\sqsubseteq }(B)((h \times k)(R))) \,. \end{aligned}$$

The latter inclusion in turn follows from the following two facts:

- $(h \times k) (f \times g)^{-1} \subseteq (f' \times g')^{-1} (Bh \times Bk)$ (pointwise inclusion). This is a straightforward computation: let $S \subseteq BX \times BY$, and suppose $(f(x), g(y)) \in S$; we need to prove that $(h(x),h(y)) \in (f' \times g')^{-1} (Bh \times Bk)(S)$. Indeed, we have $((Bh)(f(x)),(Bk)(g(y))) \in (Bh \times Bk)(S)$, so we have $(f'(h(x)), k'(g(y))) \in (Bh \times Bk)(S)$ (since *h* and *k* are homomorphisms), as desired.
- $(Bh \times Bk) (\mathsf {Rel}_{\sqsubseteq }(B)(R)) \subseteq \mathsf {Rel}_{\sqsubseteq }(B)((h \times k)(R))$ is shown concretely in [12, Lemma 4.2].

$$\square $$

Pointwise inequality of coalgebras implies pointwise similarity of coinductive extensions:

### Lemma 4.6

Let $(B, \sqsubseteq )$ be an ordered functor, and let *f* and *g* be *B*-coalgebras on a common carrier *X*. If $f \mathrel {\sqsubseteq _{BX}} g$ then $f^\infty \mathrel {\lesssim _{B^\infty X}} g^\infty$.

### Proof

Suppose $f \mathrel {\sqsubseteq _{BX}} g$. This is equivalent to $(f \times g)(\varDelta _X) \subseteq {\sqsubseteq _{BX}}$. Since $\sqsubseteq _{BX}$ is reflexive, we have $\sqsubseteq _{BX} \subseteq {(\sqsubseteq _{BX} \circ \varDelta _{BX} \circ \sqsubseteq _{BX})}$. Further $\varDelta _{BX} = \mathsf {Rel}(B)(\varDelta _X)$ by Lemma 4.1 (1), so that $\sqsubseteq _{BX} \subseteq {(\sqsubseteq _{BX} \circ \mathsf {Rel}(B)(\varDelta _X) \circ \sqsubseteq _{BX})} = \mathsf {Rel}_{\sqsubseteq }(B)(\varDelta _X)$. Combined with the assumption, this yields $(f \times g)(\varDelta _X) \subseteq \mathsf {Rel}_{\sqsubseteq }(B)(\varDelta _X)$, so $\varDelta _X$ is a simulation between *f* and *g*. By Lemma 4.2 this implies that $x \lesssim x$ for any $x \in X$, where $\lesssim$ is the similarity order between $(X, \langle f, \mathsf {id}\rangle )$ and $(X, \langle g, \mathsf {id}\rangle )$ induced by $(B \times X, {\widetilde{\sqsubseteq }})$. Notice that $f^\infty$ and $g^\infty$ are coalgebra homomorphisms from $\langle f, \mathsf {id}\rangle$ and $\langle g, \mathsf {id}\rangle$ respectively into the cofree coalgebra $(B^\infty X, \langle \theta , \epsilon \rangle )$, so by Lemma 4.5 we have $(f^\infty \times g^\infty )(\varDelta _X) \subseteq {\lesssim _{B^\infty X}}$. $\square$

Recall from Sect. 2 that any *B*-homomorphism yields a $B^\infty$-homomorphism between coinductive extensions. A similar fact holds for inequalities.

### Lemma 4.7

Let $(B, \sqsubseteq )$ be an ordered functor where *B* preserves weak pullbacks, and let $f :X \rightarrow BX$, $g :Y \rightarrow BY$ and $h :X \rightarrow Y$.

1. If $$Bh \circ f \sqsubseteq _{BY} g \circ h$$ then $$B^\infty h \circ f^\infty \lesssim _{B^\infty Y} g^\infty \circ h$$.
2. If $$Bh \circ f \sqsupseteq _{BY} g \circ h$$ then $$B^\infty h \circ f^\infty \gtrsim _{B^\infty Y} g^\infty \circ h$$.

### Proof

The assumption in item 1. is equivalent to: $(f(x),g(h(x)) \in (\mathsf {Graph}(Bh) \circ {\sqsubseteq _{BY}})$ for all $x \in X$. Thus $(f(x), g(h(x)) \in ({\sqsubseteq _{BX}} \circ \mathsf {Graph}(Bh) \circ {\sqsubseteq _{BY}})$ since $\sqsubseteq _{BX}$ is reflexive. We have $\mathsf {Graph}(Bh) = \mathsf {Rel}(B)(\mathsf {Graph}(h))$ by Lemma 4.1 (5) and the assumption that *B* preserves weak pullbacks, so by the above, $\mathsf {Graph}(h)$ is a simulation between *f* and *g*. By Lemma 4.2 it follows that $x \lesssim h(x)$, where $\lesssim$ is the similarity order between $\langle f, h \rangle :X \rightarrow BX \times Y$ and $\langle g, \mathsf {id}\rangle :Y \rightarrow BY \times Y$ induced by $(B \times Y, {\widetilde{\sqsubseteq }})$.

Next, observe that from the following commutative diagram it follows that $B^\infty h \circ f^\infty$ is a $B\times Y$-coalgebra homomorphism:The upper left square commutes by definition of $f^\infty$, the upper right by naturality of $\theta$ and $\epsilon$, and the rest trivially. Since $g^\infty$ is a coalgebra homomorphism as well (from $\langle g, \mathsf {id}\rangle$ to $(B^\infty Y, \langle \theta _Y, \epsilon _Y \rangle )$), by the fact that coalgebra homomorphisms preserve similarity (Lemma 4.5) we obtain $B^\infty h \circ f^\infty \lesssim _{B^\infty Y} g^\infty \circ h$ as desired.

For item 2. by assumption we have $g(h(x)) \sqsubseteq _{BY} Bh(f(x))$ for all $x \in X$, so $(g(h(x)), f(x)) \in ({\sqsubseteq _{BY}} \circ \mathsf {Graph}(Bh)^\mathsf {op})$, which implies $(g(h(x)), f(x)) \in ({\sqsubseteq _{BY}} \circ \mathsf {Graph}(Bh)^\mathsf {op}\circ {\sqsubseteq _{BX}})$. But we have

$$\begin{aligned} \mathsf {Graph}(Bh)^\mathsf {op}= (\mathsf {Rel}(B)(\mathsf {Graph}(h)))^\mathsf {op}= \mathsf {Rel}(B)(\mathsf {Graph}(h)^\mathsf {op}) \end{aligned}$$

by Lemma 4.1 (item 4 and 5 respectively), so by the above, $\mathsf {Graph}(h)^\mathsf {op}$ is a simulation between *g* and *f*. Similar to the above case, applying homomorphisms $g^\infty$ and $B^\infty h \circ f^\infty$ yields the desired result. $\square$

## Monotone biGSOS specifications

### Definition 5.1

A biGSOS specification $\rho :\varSigma B^\infty \Rightarrow B \varSigma ^*$ is *monotone* if the restriction of $\rho _X \times \rho _X$ to $\mathsf {Rel}(\varSigma )(\lesssim _{B^\infty X})$ corestricts to $\sqsubseteq _{B\varSigma ^*X}$, for any set *X*.

If $\varSigma$ represents a signature, then monotonicity can be conveniently restated as follows (c.f. [6], where monotone GSOS is characterised in a similar way). For every operator $\sigma$:

$$\begin{aligned} \frac{b_1 \lesssim _{B^\infty X} c_1 \quad \ldots \quad b_n \lesssim _{B^\infty X} c_n}{\rho _X(\sigma (b_1, \ldots , b_n)) \sqsubseteq _{B \varSigma ^* X} \rho _X(\sigma (c_1, \ldots , c_n))} \end{aligned}$$

for every set *X* and every $b_1, \ldots , b_n, c_1, \ldots , c_n \in B^\infty X$. Thus, in a monotone specification, if $c_i$ simulates $b_i$ for each *i*, then the behaviour of $\sigma (b_1, \ldots , b_n)$ is “less than” the behaviour of $\sigma (c_1, \ldots , c_n)$.

### Example 5.2

In the case of an ntree specification for labelled transition systems, if there are no negative premises then the induced biGSOS specification (Example 3.6) is monotone.

To see this, suppose that for all *i*, we have $x_i \models _h b_i$ (notation of Example 3.6), and $b_i \lesssim _{B^\infty X} c_i$. By definition of $\lesssim _{B^\infty X}$ (cf. Example 4.3) and $\models _h$, and the absence of negative premises, it follows that there is a map $k :V \rightarrow B^\infty X$ from the set of variables occuring in the premises of the rules (notation of Example 3.6), such that $\epsilon _X \circ k = \epsilon _X \circ h$ (i.e., they agree on node labels), and for all *i*: $x_i \models _k c_i$. It follows that $\rho _X(\sigma (b_1, \ldots , b_n)) \sqsubseteq _{B \varSigma ^* X} \rho _X(\sigma (c_1, \ldots , c_n))$, as needed.

Notice that the example specification in the introduction consisting of rules (2) and (3), which does not have a model, is not monotone. This is no coincidence: every monotone biGSOS specification has a model, if $B\varSigma ^*\emptyset$ is a pointed DCPO, as we will see next. In fact, the proper canonical choice is the *least* model, corresponding to behaviour obtained in finitely many proof steps.

### Models of monotone specifications

Let $\rho$ be a monotone biGSOS specification. Suppose $B\varSigma ^*\emptyset$ is a pointed DCPO. Then the set of coalgebras $\mathsf {coalg}(B)_{\varSigma ^*\emptyset } = \{f \mid f :\varSigma ^*\emptyset \rightarrow B\varSigma ^*\emptyset \}$, ordered pointwise, is a pointed DCPO as well.

Consider the function $\varphi :\mathsf {coalg}(B)_{\varSigma ^*\emptyset } \rightarrow \mathsf {coalg}(B)_{\varSigma ^*\emptyset }$, defined as follows:

7 $$\begin{aligned} \varphi (f) = B\mu _\emptyset \circ \rho _{\varSigma ^*\emptyset } \circ \varSigma f^\infty \circ \iota _\emptyset ^{-1} \end{aligned}$$

where $\iota _\emptyset :\varSigma \varSigma ^* \emptyset \rightarrow \varSigma ^* \emptyset$ is the initial $\varSigma$-algebra. Since $\iota _\emptyset$ is an isomorphism (Sect. 2.1), a function *f* is a fixed point of $\varphi$ if and only if it is a supported model of $\rho$ (Eq. 6). We are interested in the *least* supported model. To show that it exists, since $\mathsf {coalg}(B)_{\varSigma ^*\emptyset }$ is a pointed DCPO, it suffices to show that $\varphi$ is monotone.

#### Lemma 5.3

The function $\varphi$ is monotone.

#### Proof

Suppose $f,g :\varSigma ^*\emptyset \rightarrow B\varSigma ^*\emptyset$ and $f \sqsubseteq _{B\varSigma ^*\emptyset } g$. By Lemma 4.6, we have $f^\infty \lesssim _{B^\infty \varSigma ^* \emptyset } g^\infty$. By Lemma 4.1 (6) we derive

$$\begin{aligned} \varSigma f^\infty \mathrel {\mathsf {Rel}(\varSigma )(\lesssim _{B^\infty \varSigma ^* \emptyset })} \varSigma g^\infty \end{aligned}$$

and now the result follows by monotonicity of $\rho$ (assumption) and monotonicity of $B \mu _\emptyset$ (*B* is ordered). $\square$

#### Corollary 5.4

If $B\varSigma ^*\emptyset$ is a pointed DCPO and $\rho$ is a monotone biGSOS specification, then $\rho$ has a least supported model.

The condition of the Corollary is satisfied if *B* is of the form $B = G + 1$ (c.f. Example 4.4), that is, $B = G + 1$ for some functor *G* (where the element in the singleton 1 is interpreted as the least element of the pointed DCPO). Consider, as an example, the functor $BX = A \times X + 1$ of finite and infinite streams over *A*. Any specification that does not mention termination (i.e., a specification for the functor $GX = A \times X$) yields a monotone specification for *B*.

#### Example 5.5

Consider the following specification (in terms of rules) for the functor $BX = \mathbb {N} \times X + 1$ of (possibly terminating) stream systems over the natural numbers. It specifies a unary operator $\sigma$, a binary operator $\oplus$, infinitely many unary operators $m \otimes -$ (one for each $m \in \mathbb {N}$), and constants $ones , pos$, *c*:

$$\begin{aligned} \frac{x \xrightarrow {n} x' \qquad x' \xrightarrow {m} x''}{\sigma (x) \xrightarrow {n} n \otimes (m \otimes \sigma (x''))} \qquad \frac{x \xrightarrow {n} x' \qquad y \xrightarrow {m} y'}{x \oplus y \xrightarrow {n+m} x' \oplus y'} \qquad \frac{x \xrightarrow {n} x'}{m \otimes x \xrightarrow {m \times n} m \otimes x'} \end{aligned}$$

$$\begin{aligned} \frac{}{ ones \xrightarrow {1} ones } \qquad \frac{}{ pos \xrightarrow {1} ones \oplus pos } \qquad \frac{}{c \xrightarrow {1} \sigma (c)} \end{aligned}$$

where $+$ and $\times$ denote addition and multiplication of natural numbers, respectively. The rules for $\oplus$ and $\otimes$ define standard pointwise addition and multiplication by a constant, respectively. The operator $\sigma$ is slightly more curious; of interest here is that it requires lookahead, and is therefore not a GSOS rule. In fact, the rule for $\sigma$ is GSOS nor coGSOS, since it uses both lookahead and a complex conclusion. By the above Corollary, it has a model. The coinductive extension maps $pos$ to the increasing stream of positive integers, and $\sigma ( pos )$ is the stream $(1,6,120, \ldots ) = (1!, 3!, 5!, \ldots )$. But *c* does not represent an infinite stream, since $\sigma (c)$ is undefined.

The case of *labelled transition systems* is a bit more subtle. The problem is that $(\mathcal {P}_f\varSigma ^* \emptyset )^A$ and $(\mathcal {P}_c\varSigma ^* \emptyset )^A$ are not DCPOs, in general, whereas the functor $(\mathcal {P}-)^A$ does not have a cofree comonad. However, if the set of closed terms $\varSigma ^* \emptyset$ is countable, then $(\mathcal {P}_c\varSigma ^* \emptyset )^A$ is a pointed DCPO, and thus Corollary 5.4 applies. The specification in Example 3.7 can be viewed as a specification for the functor $(\mathcal {P}_c-)^A$, and it has a countable set of terms. Therefore it has, by the Corollary, a least supported model. In this model, the behaviour of $\sigma (t)$ is empty, for any $t \in \varSigma ^* \emptyset$.

## Distributive laws for biGSOS specifications

In the previous section we have seen how to construct a least supported model of a monotone biGSOS specification, as the least fixed point of a certain monotone function. In the present section we show that, given a monotone biGSOS specification, the construction of a least model generalises to a *lifting* of the free monad $\varSigma ^*$ to the category of *B*-coalgebras. It then immediately follows that there exists a canonical distributive law of the monad $\varSigma ^*$ over the comonad $B^\infty$, and that the (unique) operational model of this distributive law corresponds to the least supported model as constructed above.

In order to proceed we define a $\mathsf {DCPO}_\bot$-*ordered functor* as an ordered functor (Sect. 4) where $\mathsf {PreOrd}$ is replaced by $\mathsf {DCPO}_\bot$. Below we assume that $(B, \sqsubseteq )$ is $\mathsf {DCPO}_\bot$-ordered, and $\varSigma$ and *B* are as before (having a free monad and cofree comonad respectively). Throughout this section we assume a monotone biGSOS specification $\rho :\varSigma B^\infty \Rightarrow B\varSigma ^*$.

### Example 6.1

A general class of functors that are $\mathsf {DCPO}_\bot$-ordered are those of the form $B + 1$, where the singleton 1 is interpreted as the least element and all other distinct elements are incomparable (see Example 4.4). Another example is the functor $(\mathcal {P}-)^A$ of labelled transition systems with arbitrary branching, but this example can not be treated here because there exists no cofree comonad for it. The case of labelled transition systems is treated in Sect. 7.

Let $\mathsf {coalg}(B)_{\varSigma ^* X}$ be the set of *B*-coalgebras with carrier $\varSigma ^* X$, pointwise ordered as a DCPO by the order on *B*. The lifting of $\varSigma ^*$ to $\mathsf {coalg}(B)$ that we are about to define maps a coalgebra $c :X \rightarrow BX$ to the least coalgebra $\overline{c} :\varSigma ^*X \rightarrow B\varSigma ^*X$, w.r.t. the above order on $\mathsf {coalg}(B)_{\varSigma ^*X}$, making the following diagram commute.8Equivalently, $\overline{c}$ is the least fixed point of the operator

$$\begin{aligned} \begin{array}{rcl} \varphi _c &{}:&{}\mathsf {coalg}(B)_{{\varSigma ^* X}} \rightarrow \mathsf {coalg}(B)_{{\varSigma ^* X}} \\ &{}&{}f \mapsto [B\mu _X \circ \rho _{\varSigma ^*\emptyset } \circ \varSigma f^\infty , B\eta _X \circ c] \circ [\iota _X, \eta _X]^{-1} \,. \end{array} \end{aligned}$$

Following the proof of Lemma 5.3 it is easy to verify:

### Lemma 6.2

For any $c :X \rightarrow BX$, the function $\varphi _c$ is monotone.

For the lifting of $\varSigma ^*$, we need to show that the above construction preserves coalgebra morphisms.

### Theorem 6.3

The functor $\overline{\varSigma ^*} :\mathsf {coalg}(B) \rightarrow \mathsf {coalg}(B)$ defined by

$$\begin{aligned} \overline{\varSigma ^*}(X, c) = (\varSigma ^*X, \overline{c}) \qquad \text { and } \qquad \overline{\varSigma ^*}(h) = \varSigma ^*h \end{aligned}$$

is a lifting of the functor $\varSigma ^*$.

### Proof

Let (*X*, *c*) and (*Y*, *d*) be $B\varSigma ^*$-coalgebras. We need to prove that, if $h :X \rightarrow Y$ is a coalgebra homomorphism from *c* to *d*, then $\varSigma ^*h$ is a homomorphism from $\overline{c}$ to $\overline{d}$.

The proof is by transfinite induction on the iterative construction of $\overline{c}$ and $\overline{d}$ as limits of the ordinal-indexed initial chains of $\varphi _c$ and $\varphi _d$ respectively. For the limit (and base) case, given a (possibly empty) directed family of coalgebras $f_i :\varSigma ^* X \rightarrow B\varSigma ^*X$ and another directed family $g_i :\varSigma ^* Y \rightarrow B\varSigma ^*Y$, such that $B \varSigma ^* h \circ f_i = g_i \circ \varSigma ^* h$ for all *i*, we have $B\varSigma ^* h \circ \bigvee _i f_i = \bigvee _i (B\varSigma ^* h \circ f_i) = \bigvee _i (g_i \circ \varSigma ^* h) = (\bigvee _i g_i) \circ \varSigma ^* h$ by continuity of $B\varSigma ^* h$ and assumption.

Let $f :\varSigma ^*X \rightarrow B\varSigma ^*X$ and $g :\varSigma ^* Y \rightarrow B\varSigma ^* Y$ be such that $B\varSigma ^*h \circ f = g \circ \varSigma ^* h$. To prove: $B\varSigma ^*h \circ \varphi _c(f) = \varphi _d(g) \circ \varSigma ^*h$, i.e., commutativity of the outside of:From left to right, the first square commutes by naturality of $[\iota ,\eta ]$ (and the fact that it is an isomorphism), the second by assumption that $\varSigma ^*h$ is a *B*-coalgebra homomorphism from *f* to *g* (and therefore a $B^\infty$-coalgebra homomorphism) and the assumption that *h* is a coalgebra homomorphism from *c* to *d*, the third by naturality of $\rho$, and the fourth by naturality of $\mu$ and $\eta$. $\square$

We show that the (free) *monad*$(\varSigma ^*,\eta ,\mu )$ lifts to $\mathsf {coalg}(B)$. This is the heart of the matter. The main proof obligation is to show that $\mu _X$ is a coalgebra homomorphism from $\overline{\varSigma ^*}(\overline{\varSigma ^*}(X,c))$ to $\overline{\varSigma ^*}(X,c)$, for any *B*-coalgebra (*X*, *c*).

### Theorem 6.4

If *B* preserves weak pullbacks, then the functor $\overline{\varSigma ^*}$ extends to a monad $(\overline{\varSigma ^*},\eta ,\mu )$ on $\mathsf {coalg}(B)$, which is a lifting of the free monad $(\varSigma ^*,\eta ,\mu )$.

### Proof

Let (*X*, *c*) be a $B\varSigma ^*$-coalgebra. We need to show that $\eta _X :X \rightarrow \varSigma ^* X$ is a coalgebra homomorphism from (*X*, *c*) to $\overline{\varSigma ^*}(X,c)$, and $\mu _X :\varSigma ^*\varSigma ^*X \rightarrow \varSigma ^*X$ is a coalgebra homomorphism from $\overline{\varSigma }(\overline{\varSigma }(X,c))$ to $\overline{\varSigma }(X,c)$:For $\eta _X$, this is immediate from the definition of $\overline{c}$. For $\mu _X$, we split the proof in two inequalities, both established by transfinite induction. First we prove $B\mu _X \circ \overline{\overline{c}} \sqsubseteq _{B\varSigma ^*X} \overline{c} \circ \mu _X$. Suppose $B\mu _X \circ f_i \sqsubseteq _{B\varSigma ^*X} \overline{c} \circ \mu _X$ for a (possibly empty) directed family of coalgebras $f_i :\varSigma ^* \varSigma ^* X \rightarrow B\varSigma ^*\varSigma ^*X$. Then

$$\begin{aligned} B \mu _X \circ \bigvee _i f_i = \bigvee _i (B\mu _X \circ f_i) \sqsubseteq _{B\varSigma ^*X} \overline{c} \circ \mu _X \end{aligned}$$

by assumption and since $B\mu _X$ is continuous (*B* is $\mathsf {DCPO}_\bot$-ordered).

Now suppose $B\mu _X \circ f \sqsubseteq _{B\varSigma ^*X} \overline{c} \circ \mu _X$ for some $f :\varSigma ^* \varSigma ^* X \rightarrow B\varSigma ^*\varSigma ^*X$. We must show that $B\mu _X \circ \varphi _{\overline{c}}(f) \sqsubseteq _{B\varSigma ^*X} \overline{c} \circ \mu _X$. Applying Lemma 4.7 to the assumption yields $B^\infty \mu _X \circ f^\infty \lesssim _{B^\infty \varSigma ^*X} (\overline{c})^\infty \circ \mu _X$. Applying Lemma 4.1 (6) yields

$$\begin{aligned} {\varSigma (B^\infty \mu _X \circ f^\infty )}~ \mathsf {Rel}(\varSigma )(\lesssim _{B^\infty \varSigma ^*X})~ {\varSigma ((\overline{c})^\infty \circ \mu _X)} \,. \end{aligned}$$

Since $\rho$ is monotone, and $B\mu _X$ as well, we obtain

9 $$\begin{aligned} B \mu _X \circ \rho _{\varSigma ^*X} \circ \varSigma (B^\infty \mu _X \circ f^\infty ) \sqsubseteq _{B\varSigma ^*X} B \mu _X \circ \rho _{\varSigma ^*X} \circ \varSigma ((\overline{c})^\infty \circ \mu _X) \,. \end{aligned}$$

The following commutes by naturality of $\rho$ and a monad axiom:10Combined with (9), this yields

11 $$\begin{aligned} B \mu _X \circ B \mu _{\varSigma ^*X} \circ \rho _{\varSigma ^* \varSigma ^* X} \circ \varSigma f^\infty \sqsubseteq _{B\varSigma ^*X} B \mu _X \circ \rho _{\varSigma ^*X} \circ \varSigma (\overline{c})^\infty \circ \varSigma \mu _X \,. \end{aligned}$$

Now consider the following diagram:The left-most and right-most parts commute; by definition of $\varphi$ and since $\varphi _c(\overline{c}) = \overline{c}$. For the upper square, one uses that $[\iota _X, \eta _X]$ and $[\iota _{\varSigma ^*X}, \eta _{\varSigma ^* X}]$ are isomorphisms, and proves that for both components of $\varSigma \varSigma ^* \varSigma ^* X + \varSigma ^* X$, the two paths to $\varSigma ^*$ (obtained by inverting the iso) commute; for the left component this is because $\mu _X$ is an algebra homomorphism from $\iota _{\varSigma ^*X}$ to $\iota _X$, for the right component since $\mu _X \circ \eta _{\varSigma ^*X} = \mathsf {id}= [\iota _X,\eta _X] \circ [\iota _X,\eta _X]^{-1}$.

Now, proving that $B\mu _X \circ \varphi _{\overline{c}}(f) \sqsubseteq _{B\varSigma ^*X} \overline{c} \circ \mu _X$, by the above diagram reduces to Eq. (11)—which we already have—and

12 $$\begin{aligned} B \mu _X \circ B\eta _{\varSigma ^* X} \circ \overline{c} \sqsubseteq _{B\varSigma ^*X} [B\mu _X \circ \rho _{\varSigma ^*\emptyset } \circ \varSigma (\overline{c})^\infty , B \eta _X \circ c] \circ [\iota _X, \eta _X]^{-1} (= \varphi _c(\overline{c})) \quad \end{aligned}$$

which holds since $B\mu _X \circ B\eta _{\varSigma ^* X} = \mathsf {id}$ (a monad axiom), and $\overline{c} = \varphi _c(\overline{c})$. This concludes the proof for the successor step.

For the other inequality, i.e., $B\mu _X \circ \overline{\overline{c}} \sqsupseteq _{B\varSigma ^*X} \overline{c} \circ \mu _X$, one proves again by transfinite induction that $B\mu _X \circ \overline{\overline{c}} \sqsupseteq _{B\varSigma ^*X} f \circ \mu _X$ for any $f :\varSigma ^*X \rightarrow B\varSigma ^*X$ in the chain approximating $\overline{c}$. The limit case is straightforward. For the successor step, assume $B\mu _X \circ \overline{\overline{c}} \sqsupseteq _{B\varSigma ^*X} f \circ \mu _X$; we must prove that $B\mu _X \circ \overline{\overline{c}} \sqsupseteq _{B\varSigma ^*X} \varphi _c(f) \circ \mu _X$.

We apply Lemma 4.7 to the assumption, to obtain $B^\infty \mu _X \circ (\overline{\overline{c}})^\infty \gtrsim _{B^\infty \varSigma ^* X} f^\infty \circ \mu _X$. Similar to the previous case, we derive

13 $$\begin{aligned} B\mu _X \circ B\mu _{\varSigma ^*X} \circ \rho _{\varSigma ^* \varSigma ^* X} \circ \varSigma (\overline{\overline{c}})^\infty \sqsupseteq _{B\varSigma ^*X} B\mu _X \circ \rho _{\varSigma ^* X} \circ \varSigma (f^\infty \circ \mu _X)\,. \end{aligned}$$

And we consider the following diagram:The left-most and right-most parts commute; by definition of $\varphi$ and since $\varphi _{\overline{c}}(\overline{\overline{c}}) = \overline{\overline{c}}$. Similar to the previous case, the upper square commutes, and because of (13) the only remaining proof obligation is:

$$\begin{aligned} \overline{c} \sqsupseteq _{B\varSigma ^*X} [B\mu _X \circ \rho _{\varSigma ^*\emptyset } \circ \varSigma f^\infty , B\eta _X \circ c] \circ [\iota _X, \eta _X]^{-1} (= \varphi _c(f)) \end{aligned}$$

which holds: we have $\overline{c} \sqsupseteq _{B\varSigma ^*X} f$ by assumption, hence $\varphi _c(\overline{c}) \sqsupseteq _{B\varSigma ^*X} \varphi _c(f)$ since $\varphi _c$ is monotone, and combined with $\varphi _c(\overline{c}) = \overline{c}$ this gives the desired result. $\square$

The lifting gives rise to a distributive law of monad over comonad.

### Theorem 6.5

Let $\rho :\varSigma B^\infty \Rightarrow B \varSigma ^*$ be a monotone biGSOS specification, where *B* is $\mathsf {DCPO}_\bot$-ordered and preserves weak pullbacks. There exists a distributive law $\lambda :\varSigma ^* B^\infty \Rightarrow B^\infty \varSigma ^*$ of the free monad $\varSigma ^*$ over the cofree comonad $B^\infty$ such that the operational model of $\lambda$ is the least supported model of $\rho$.

### Proof

By Theorem 6.4, we obtain a lifting of $(\varSigma ^*,\eta ,\mu )$ to $\mathsf {coalg}(B)$. As explained in Sect. 2.3, such a lifting corresponds uniquely to a distributive law of the desired type. The operational model of $\lambda$ can be obtained by applying the lifting to the unique coalgebra $! :\emptyset \rightarrow B\emptyset$. But that coincides, by definition of the lifting, with the least supported model as defined in Sect. 5. $\square$

It follows from the general theory of bialgebras that the unique coalgebra morphism from the least supported model to the final coalgebra is an algebra homomorphism, i.e., behavioural equivalence on the least supported model of a monotone biGSOS specification is a congruence.

At the end of Sect. 3, we recalled that in general it is undecidable whether a given biGSOS specification extends uniquely to a distributive law. Above, we have shown how to construct a distributive law for a *monotone* biGSOS specification, in terms of a lifting. We next show that the constructed distributive law indeed extends the original biGSOS specification.

### Theorem 6.6

The distributive law $\lambda$ constructed in Theorem 6.5 extends the original monotone biGSOS specification $\rho$.

### Proof

We first recall how the distributive law $\lambda$ is obtained from the monad lifting $\overline{\varSigma ^*}$. To this end, let $\overline{\theta _X} :\varSigma ^* X \rightarrow B\varSigma ^* X$ be the coalgebra obtained by applying the lifting $\overline{\varSigma ^*}$ to the coalgebra $\theta _X :B^\infty \rightarrow BB^\infty$. The distributive law $\lambda$ is characterised, on a component *X*, by finality, as the unique map making the following diagram commute:14Now, consider the following diagram. We will prove that the entire diagram commutes, which implies that $\lambda$ is an extension of $\rho$.The upper crescent commutes by a comonad axiom, the left by definition of $\kappa$ and the bottom by definition of $\nu$. The rectangle on the left in the middle commutes by definition of $\overline{\theta _X}$, see (8). The squares on the bottom row commute by (14). Most of the remaining parts commute by naturality; except the upper square on the left. For that, observe first that $\eta _{B^\infty X}$ is a coalgebra morphism by Theorem 6.4, as on the left below:Since coinductive extensions preserve homomorphisms, the diagram on the right commutes; finally, we use that $\theta _X^\infty = \delta _X$. $\square$

*Labelled transition systems* The results above do not apply to labelled transition systems. The problem is that the cofree comonad for the functor $(\mathcal {P}-)^A$ does not exist. A first attempt would be to restrict to the finitely branching transition systems, i.e., coalgebras for the functor $(\mathcal {P}_f-)^A$. But this functor is not $\mathsf {DCPO}_\bot$-ordered, and indeed, contrary to the case of GSOS and coGSOS, even with a finite biGSOS specification one can easily generate a least model with infinite branching, so that a lifting as in the previous section can not exist.

### Example 6.7

Consider the following specification on (finitely branching) labelled transition systems, involving a unary operator $\sigma$ and a constant *c*:

$$\begin{aligned} \frac{}{c \xrightarrow {a} \sigma (c)} \qquad \frac{}{\sigma (x) \xrightarrow {a} \sigma (\sigma (x))} \qquad \frac{x \xrightarrow {a} x' \xrightarrow {a} x'' \xrightarrow {a} x'''}{\sigma (x) \xrightarrow {a} x'''} \end{aligned}$$

The left rule for $\sigma$ constructs an infinite chain of transitions from $\sigma (x)$ for any *x*, so in particular for $\sigma (c)$. The right rule takes the transitive closure of transitions from $\sigma (c)$, so in the least model there are infinitely many transitions from $\sigma (c)$.

The model in the above example has countable branching. One might ask whether it can be adapted to generate uncountable branching, i.e., that we can construct a biGSOS specification for the functor $(\mathcal {P}_c-)^A$, such that the model of this specification would feature uncountable branching. However, as it turns out, this is not the case, at least if we assume $\varSigma$ to be a polynomial functor (a countable coproduct of finite products, modelling a signature with countably many operations each of finite arity), and the set of labels *A* to be countable. This is shown more generally in the next section.

## Liftings for countably accessible functors

In the previous section, we have seen that one of the most important instances—the case of labelled transition systems—does not meet the necessary assumptions for our construction of a distributive law, because of size issues: the functors in question either do not have a cofree comonad, or are not DCPO-ordered. In the current section, we solve this problem by showing that, if both functors $B, \varSigma$ are reasonably well-behaved, then it suffices to have a DCPO-ordering of *B* only on *countable* sets. The general approach is to first construct a lifting of the free monad to the category of coalgebras with a countable carrier, and subsequently extend it to a lifting to the category of all coalgebras.

To state the assumption more precisely, let $\mathsf {cSet}$ be the full subcategory of countable sets, with inclusion $I :\mathsf {cSet}\rightarrow \mathsf {Set}$. We assume that $(B,\sqsubseteq )$ is an ordered functor on $\mathsf {Set}$, and that its restriction to countable sets is $\mathsf {DCPO}_\bot$-ordered:This is a weaker assumption than in Sect. 6: before, every set *BX* was assumed to be a pointed DCPO, whereas here, they only need to be pointed DCPOs when *X* is countable (and just a preorder otherwise).

### Example 7.1

The functor $(\mathcal {P}_c-)^A$ coincides with the $\mathsf {DCPO}_\bot$-ordered functor $(\mathcal {P}-)^A$ when restricted to countable sets, hence it satisfies the above assumption. Notice that $(\mathcal {P}_c-)^A$ is not $\mathsf {DCPO}_\bot$-ordered. The functor $(\mathcal {P}_f-)^A$ does not satisfy the above assumption.

The functor $(\mathcal {M}-)^A$, for the complete monoid $\mathbb {R}^+ \cup \{\infty \}$ (Example 2.3), is ordered as a complete lattice [25], so also $\mathsf {DCPO}_\bot$-ordered. Similar to the above, the functor $(\mathcal {M}_c -)^A$ is $\mathsf {DCPO}_\bot$-ordered when restricted to countable sets, i.e., satisfies the above assumption.

We define $\mathsf {coalg}_c(B)$ to be the full subcategory of *B*-coalgebras whose carrier is a countable set, with inclusion $\overline{I} :\mathsf {coalg}_c(B) \rightarrow \mathsf {coalg}(B)$. The associated forgetful functor is denoted by $U :\mathsf {coalg}_c(B) \rightarrow \mathsf {cSet}$.

The pointed DCPO structure on each *BX*, for *X* countable, suffices to carry out the fixed point constructions from the previous sections for coalgebras over countable sets, if we assume that $\varSigma ^*$ preserves countable sets. Notice that the (partial) order on the functor *B* is still necessary to define the simulation order on $B^\infty X$, and hence speak about monotonicity of biGSOS specifications. The proof of the following theorem is essentially the same as in the previous section.

### Theorem 7.2

Suppose $\varSigma ^*$ preserves countable sets, and *B* is an ordered functor which preserves weak pullbacks and whose restriction to $\mathsf {cSet}$ is $\mathsf {DCPO}_\bot$-ordered. Let $(\varSigma ^*_c,\eta ^c,\mu ^c)$ be the restriction of $(\varSigma ^*,\eta ,\mu )$ to $\mathsf {cSet}$. Any monotone biGSOS specification $\rho :\varSigma B^\infty \Rightarrow B\varSigma ^*$ gives rise to a lifting $(\overline{\varSigma ^*}_c, \overline{\eta }^c, \overline{\mu }^c)$ of the monad $(\varSigma ^*_c,\eta ^c,\mu ^c)$ to $\mathsf {coalg}_c(B)$.

The main result of this section is that, under certain additional conditions on *B* and $\varSigma ^*$, the above lifting extends to a lifting of the monad $\varSigma ^*$ from $\mathsf {Set}$ to $\mathsf {coalg}(B)$, and hence a distributive law of the monad $\varSigma ^*$ over the cofree comonad $B^\infty$ (Theorem 7.5). It relies on the fact that, under certain conditions, we can present every coalgebra as a (filtered) colimit of coalgebras over countable sets.

We use the theory of locally (countably, i.e., $\omega _1$-) presentable categories and (countably) accessible categories. For now, we only recall a concrete characterisation of when a functor on $\mathsf {Set}$ is countably accessible, since this will be assumed both for *B* and $\varSigma ^*$ later on. For details, see [3] and the appendix; the latter also contains the proofs of the results below.

On $\mathsf {Set}$, a functor $B :\mathsf {Set}\rightarrow \mathsf {Set}$ is *countably accessible* if for every set *X* and element $x \in BX$, there is an injective function $i :Y \rightarrow X$ from a finite set *Y* and an element $y \in BY$ such that $Bi(y) = x$. Intuitively, such functors are determined by how they operate on countable sets.

### Example 7.3

Any finitary functor is countably accessible. Further, the functors $(\mathcal {P}_c-)^A$ and $(\mathcal {M}_c -)^A$ (c.f. Example 7.1) are countably accessible if *A* is countable.

A functor is called *strongly countably accessible* if it is countably accessible and additionally preserves countable sets, i.e., it restricts to a functor $\mathsf {cSet}\rightarrow \mathsf {cSet}$. We will assume this for our “syntax” functor $\varSigma ^*$. If $\varSigma$ correponds to a signature with countably many operations each of finite arity (so is a countable coproduct of finite products) then $\varSigma ^*$ is strongly countably accessible.

The central idea of obtaining a lifting to $\mathsf {coalg}(B)$ from a lifting to $\mathsf {coalg}_c(B)$ is to *extend* the monad on $\mathsf {coalg}_c(B)$ along the inclusion $\overline{I} :\mathsf {coalg}_c(B) \rightarrow \mathsf {coalg}(B)$. Concretely, a functor $T :\mathsf {Set}\rightarrow \mathsf {Set}$ extends $T_c :\mathsf {cSet}\rightarrow \mathsf {cSet}$ if there is a natural isomorphism $\alpha :IT_c {\mathop {\Rightarrow }\limits ^{\cong }} TI$. A monad $(T,\eta ,\mu )$ on $\mathsf {Set}$ extends a monad $(T_c, \eta _c, \mu _c)$ on $\mathsf {cSet}$ if $T_c$ extends *T* with some isomorphism $\alpha$ such that $\alpha \circ I\eta _c = \eta I$ and $\alpha \circ I \mu _c= \mu I \circ T\alpha \circ \alpha T_c$. This notion of extension is generalised naturally to arbitrary locally countably presentable categories. Monads on the subcategory of countably presentable objects^1^ can always be extended (Lemma B.2).

Since *B* is countably accessible, $\mathsf {coalg}(B)$ is locally countably presentable and $\mathsf {coalg}_c(B)$ is the associated category of countably presentable objects [2]. This means every *B*-coalgebra can be presented as a filtered colimit of *B*-coalgebras with countable carriers. The above lemma applies, so we can extend the monad on $\mathsf {coalg}_c(B)$ of Theorem 7.2 to a monad on $\mathsf {coalg}(B)$, resulting in Theorem 7.5 below. The latter relies on Theorem 7.4, which ensures that, doing so, we will get a lifting of the monad on $\mathsf {Set}$ that we started with.

In the remainder of this section, we will consider a slightly relaxed version of functor liftings, up to isomorphism, similar to extensions defined before. This is harmless—those still correspond to distributive laws—but since the monad on $\mathsf {coalg}(B)$ is constructed only up to isomorphism, it is more natural to work with in this setting. We say $(\overline{T}, \overline{\eta }, \overline{\mu })$ lifts $(T,\eta ,\mu )$ (up to isomorphism) if there is a natural isomorphism $\alpha :U\overline{T} \Rightarrow TU$ such that $\alpha \circ U\overline{\eta } = \eta U$ and $\alpha \circ U \overline{\mu }= \mu U \circ T\alpha \circ \alpha \overline{T}$.

### Theorem 7.4

Let $B :\mathsf {Set}\rightarrow \mathsf {Set}$ be countably accessible. Suppose $(T_c,\eta ^c,\mu ^c)$ is a monad on $\mathsf {cSet}$, which lifts to a monad $(\overline{T}_c, \overline{\eta }^c, \overline{\mu }^c)$ on $\mathsf {coalg}_c(B)$. Then

1. $(T_c,\eta ^c,\mu ^c)$ extends to $(T,\eta ,\mu )$ along $I :\mathsf {cSet}\rightarrow \mathsf {Set}$,
2. $(\overline{T}_c, \overline{\eta }^c, \overline{\mu }^c)$ extends to $(\overline{T}, \overline{\eta }, \overline{\mu })$ along $\overline{I} :\mathsf {coalg}_c(B) \rightarrow \mathsf {coalg}(B)$,
3. $(\overline{T}, \overline{\eta }, \overline{\mu })$ is a lifting (up to isomorphism) of $(T,\eta ,\mu )$.

The (functor) liftings are summarised in the following diagram. The back face represents the assumed lifting, the left and right faces commute trivially, the bottom and top faces depict the extensions of the first two items respectively, and the front face the lifting in the third item.By instantiating the above theorem with the lifting of Theorem 7.2, the third point gives us the desired lifting to $\mathsf {coalg}(B)$. In particular $T_c$ is instantiated to the restriction $\varSigma ^*_c$ of $\varSigma ^*$, which means that the extension in the first point is just $\varSigma ^*$ itself.

### Theorem 7.5

Let $\rho :\varSigma B^\infty \Rightarrow B \varSigma ^*$ be a monotone biGSOS specification, where *B* is an ordered functor whose restriction to countable sets is $\mathsf {DCPO}_\bot$-ordered, *B* is countably accessible, *B* preserves weak pullbacks, and $\varSigma ^*$ is strongly countably accessible. There exists a distributive law $\lambda :\varSigma ^* B^\infty \Rightarrow B^\infty \varSigma ^*$ of the free monad $\varSigma ^*$ over the cofree comonad $B^\infty$ such that the operational model of $\lambda$ is the least supported model of $\rho$.

As explained in Examples 7.1 and 7.3, if *B* is either $(\mathcal {P}_c -)^A$ or $(\mathcal {M}_c -)^A$ (weighted in the non-negative real numbers) with *A* countable, then it satisfies the above hypotheses (that $\mathcal {M}_c$ preserves weak pullbacks follows essentially from [10]). So the above theorem applies to labelled transition systems and weighted transition systems (of the above type) over a countable set of labels, as long as the syntax is composed of countably many operations each with finite arity. Hence, behavioural equivalence on the operational model of any biGSOS specification for such systems is a congruence.

## Future work

In this paper we provided a bialgebraic foundation of positive specification formats over ordered functors, involving rules that feature lookahead in the premises as well as complex terms in conclusions. From a practical point of view, it would be interesting to find more concrete rules formats corresponding to the abstract format of the present paper. In particular, concrete GSOS formats for weighted transition systems exist [16]; they could be a good starting point.

It is currently unclear to us whether the assumption of weak pullback preservation in the main results is necessary. This assumption is used in our proof of Lemma 4.7, which in turn is used in the proof that the free monad lifts to the category of coalgebras (Theorem 6.4). Further, in the current paper we focused on *monotone* biGSOS specifications $\rho :\varSigma B^\infty \Rightarrow B\varSigma ^*$. It could be interesting to study stronger order-theoretic conditions; for instance, *continuous* biGSOS specifications. This would require extending the DCPO-order on *B* to a DCPO-order on the cofree comonad $B^\infty$, for which the techniques of [12] may be of use. The potential advantage of continuous specifications is that they could prohibit rules which use the infinite behaviour of premises, an operationally questionable feature of coGSOS and biGSOS. We leave an investigation of continuous specifications, both at the abstract level of biGSOS and more concrete syntactic formats, for future work.

## Naturality of biGSOS induced from ntree

We show that the induced $\rho$ from an ntree specification (Example 3.6) is indeed natural. Thus, weneed to prove that for every map $f :X \rightarrow Y$:

commutes. This means concretely that for all $b_1, \ldots , b_n \in B^\infty X$ and $a \in A$: (i) for all $u \in \rho _X(\sigma (b_1, \ldots , b_n))(a)$, $\varSigma ^*f(u) \in \rho _Y(\sigma (B^\infty f(b_1), \ldots , B^\infty f(b_n)))(a)$, and (ii) for all $v \in \rho _Y(\sigma (B^\infty f(b_1), \ldots , B^\infty f(b_n)))(a)$ there is $u \in \rho _X(\sigma (b_1, \ldots , b_n))(a)$ such that $\varSigma ^*f(u) = v$.

For (i), suppose $u=\varSigma ^*(\epsilon _X \circ h)(t)$ for some *t*, *h* and rule of the form (5) s.t. $x_i \models _h b_i$ for all *i*. Since $B^\infty f$ is a *B*-coalgebra homomorphism (Example 2.2), the latter implies $x_i \models _{(B^\infty f) \circ h} B^\infty f(b_i)$ for all *i*. Further,

$$\begin{aligned} \varSigma ^* f (u) = \varSigma ^* f(\varSigma ^*(\epsilon _X \circ h))(t) = \varSigma ^* (f \circ \epsilon _X \circ h))(t) = \varSigma ^* (\epsilon _Y \circ (B^\infty f) \circ h)(t) \end{aligned}$$

by functoriality and naturality. Hence $\varSigma ^*f(u) \in \rho _Y(\sigma (B^\infty f(b_1), \ldots , B^\infty f(b_n)))(a)$ as desired.

For (ii), assume $v \in \rho _Y(\sigma (B^\infty f(b_1), \ldots , B^\infty f(b_n)))(a)$, i.e., $v = \varSigma ^*(\epsilon _Y \circ k)(t)$ for some $k :V \rightarrow B^\infty (Y)$ with $\forall i. x_i \models _k B^\infty f(b_i)$.

We define a map $h :V \rightarrow B^\infty X$, by induction on the depth *d* of the trees (recall that *V* is the set of nodes of a disjoint collection of trees), such that:

- $$(B^\infty f) \circ h = k$$, and
- for every node *x* at depth strictly below *d*, $x \xrightarrow {a} x'$ implies $h(x) \xrightarrow {a} h(x')$, and $x /\!\!\!\!\!\!\xrightarrow {a}$ implies $h(x) /\!\!\!\!\!\xrightarrow {a}$.

For $d=0$ we look at the roots $x_i$, and define $h(x_i) = b_i$. The conditions are trivially satisfied; in particular $(B^\infty f) \circ h(x_i) = k(x_i)$ since $x_i \models B^\infty f(b_i)$.

For the inductive case, suppose *h* is defined up to (and including) nodes at depth *d* satisfying the above properties, and let *x* be a node at depth *d*. If $x /\!\!\!\!\!\xrightarrow {a}$ then $k(x) /\!\!\!\!\!\xrightarrow {a}$, hence $B^\infty f(h(x)) = k(x) /\!\!\!\!\!\xrightarrow {a}$ and hence $h(x) /\!\!\!\!\!\xrightarrow {a}$, since $B^\infty f$ is a coalgebra morphism. Otherwise, suppose $x \xrightarrow {a} x'$. Then $k(x) \xrightarrow {a} k(x')$, and since $k(x) = (B^\infty f)(h(x))$ and $B^\infty f$ is a coalgebra morphism, there is a *y* such that $h(x) \xrightarrow {a} y$ and $B^\infty f(y) = k(x')$. Define $h(x') = y$. This is well-defined since $x'$ only occurs once in the tree (here we use well-foundedness), and it satisfies the requirements.

This concludes the definition of the map *h* satisfying the above properties. It follows that $x_i \models _h b_i$. Hence $\varSigma ^*(\epsilon _X \circ h)(t) \in \rho _X(\sigma (b_1, \ldots , b_n))(a)$. But $\varSigma ^*f \circ \varSigma ^*(\epsilon _X \circ h) = \varSigma ^* (f \circ \epsilon _X \circ h) = \varSigma ^* (\epsilon _Y \circ B^\infty f \circ h) = \varSigma ^*(\epsilon _Y \circ k) = v$, so we are done.

## Proofs of Section 7

In this section, we prove the results on countable presentability and accessibility that were used in Sect. 7. Parts are shown in the more general setting of $\kappa$-presentable categories; we recall a few basics here, but for understanding the proofs basic familiarity with those concepts is assumed. See [3] for an extensive treatment.

A category $\mathcal {C}$ is *locally *$\kappa$-*presentable*, for $\kappa$ a regular cardinal, if it is locally small, cocomplete, and there is a set *S* of $\kappa$-presentable objects from *C* such that every object in $\mathcal {C}$ is a $\kappa$-filtered colimit of objects from *S*. For a locally $\kappa$-presentable category $\mathcal {C}$, we denote by $\mathcal {C}_\kappa$ the full subcategory of $\kappa$-presentable objects, with the inclusion $I :\mathcal {C}_\kappa \rightarrow \mathcal {C}$.

For arbitrary categories $\mathcal {C},\mathcal {D}$ we denote the category of functors by $[\mathcal {C},\mathcal {D}]$. For $\mathcal {C}$ and $\mathcal {D}$ both locally $\kappa$-presentable categories, we say $F :\mathcal {C}\rightarrow \mathcal {C}$ is $\kappa$-accessible if it preserves $\kappa$-filtered colimits. We denote the category of such $\kappa$-accessible functors by $[\mathcal {C},\mathcal {D}]^\kappa$.

Let $\omega _1$ be the first uncountable ordinal. A category is called *locally countably presentable* if it is locally $\omega _1$-presentable, and a functor is called *countably accessible* if it is $\omega _1$-accessible. The latter coincides with the concrete notion introduced in Sect. 7.

Let $\mathcal {C},\mathcal {D}$ be locally $\kappa$-presentable categories. Let $I^* :[\mathcal {C},\mathcal {D}]^\kappa \rightarrow [\mathcal {C}_\kappa ,\mathcal {D}]$ be the functor that precomposes with the inclusion $I :\mathcal {C}_\kappa \rightarrow \mathcal {C}$. This functor has a left adjoint ${\mathsf {Lan}}_{I}$, which is the *left Kan extension (along I)*; for a functor $G :\mathcal {C}_\kappa \rightarrow \mathcal {D}$, the functor ${\mathsf {Lan}}_{I}G$ is called the left Kan extension of *G* along *I*; such a left Kan extension is always $\kappa$-accessible [21, Proposition 2.4.3]. In fact, gives rise to an adjoint *equivalence*; this is a special case of [21, Corollary 2.1.9].

15

The unit $\iota$ is given on a component *G* by the unit $\iota _G :G \rightarrow ({\mathsf {Lan}}_{I}G)I$ of the left Kan extension. Notice in particular that this unit $\iota _G$ is always an isomorphism.

### Lemma B.1

Let $\mathcal {C}$ be a locally $\kappa$-presentable category, with $I :\mathcal {C}_\kappa \rightarrow \mathcal {C}$ the subcategory of countably presentable objects. Let $F :\mathcal {C}\rightarrow \mathcal {C}$ be a $\kappa$-accessible functor, $G :\mathcal {C}_\kappa \rightarrow \mathcal {C}$ a functor and $\alpha :G \Rightarrow FI$ a natural isomorphism.

Then $(F,\alpha )$ is a left Kan extension of *G* along *I*.

### Proof

By the universal property of the Kan extension $({\mathsf {Lan}}_{I}G,\iota _G)$, there exists a unique natural transformation ${\hat{\alpha }}$ such that

Since $\iota _G$ and $\alpha$ are both iso [the former by (15)], ${\hat{\alpha }}I$ is as well. By the equivalence (15), it follows that ${\hat{\alpha }} :{\mathsf {Lan}}_{I}G \Rightarrow F$ is iso. It is now easy to show that $(F,\alpha )$ is a left Kan extension. $\square$

### Lemma B.2

Let $\mathcal {C}$ be a locally $\kappa$-presentable category, with $I :\mathcal {C}_\kappa \rightarrow \mathcal {C}$ the subcategory of countably presentable objects. Any monad $(T_\kappa , \eta ^\kappa ,\mu ^\kappa )$ on $\mathcal {C}_\kappa$ extends uniquely to a ($\kappa$-accessible) monad $(T,\eta ,\mu )$ on $\mathcal {C}$, along $I :\mathcal {C}_\kappa \rightarrow \mathcal {C}$.

### Proof

First of all, define *T* as the left Kan extension of $IT_\kappa$ along *I*, with unit $\alpha :IT_\kappa \Rightarrow TI$ [this is an isomorphism (15)]. By Lemma B.1 it follows that, for any *i*, $T^i$ is a left Kan extension of $IT_\kappa ^i$ along *I*. In particular, $T^0 = \mathsf {Id}$ and $T^2$ are Kan extensions:

Now, to define the unit and multiplication of *T*, we use the universal property of the above left Kan extensions respectively on $\alpha \circ \eta ^\kappa :I \Rightarrow TI$ and $\alpha \circ I\mu ^\kappa :IT_\kappa T_\kappa \Rightarrow TI$ to obtain a unique $\eta$ and $\mu$ making the following diagrams commute:

16

Once we show that $(T,\eta ,\mu )$ is a monad, the above diagrams assert that $(T,\eta ,\mu )$ is an extension of $(T_\kappa , \eta ^\kappa , \mu ^\kappa )$.

For the monad axioms, first consider the following diagram:

The crescent commutes by a monad axiom, the upper left part by naturality of $\alpha$, and the triangle and lower rectangle by 16. Hence $\mu I \circ T\eta I \circ \alpha = \alpha = \mathsf {id}\circ \alpha$, which means $\mu \circ T\eta = \mathsf {id}$ by the universal property of the Kan extension $(T,\alpha )$. In a similar way we obtain $\mu I \circ \eta TI \circ \alpha = \alpha$, so that $\mu \circ \eta T = \mathsf {id}$.

Finally, for the associativity consider:

which commutes by (16), a monad axiom (associativity $\mu ^\kappa$) and naturality. Hence,

$$\begin{aligned} \mu I \circ T \mu I \circ TT\alpha \circ T \alpha T_\kappa \circ \alpha T_\kappa T_\kappa = \mu I \circ \mu TI \circ TT\alpha \circ T \alpha T_\kappa \circ \alpha T_\kappa T_\kappa \, \end{aligned}$$

so that $\mu \circ T\mu = \mu \circ \mu T$, since $(T^3, TT\alpha \circ T \alpha T_\kappa \circ \alpha T_\kappa T_\kappa )$ is a left Kan extension of $IT^3$ along *I*. $\square$

We now move to countably accessible functors *B* on $\mathsf {Set}$, to prove the main lifting theorem.

### Proof of Theorem 7.4

The first two points are instances of Lemma B.2. For the second point, we use that $\mathsf {coalg}(B)$ is locally countably presentable, with $\mathsf {coalg}_c(B)$ the set of countably presentable objects, since $B :\mathsf {Set}\rightarrow \mathsf {Set}$ is a countably presentable $\mathsf {Set}$ endofunctor [2]. (This is a non-trivial result, and $\mathsf {coalg}(B)$ is not finitely presentable in general even for finitary functors *B*, as shown as well in [2].)

It remains to show that $(\overline{T},\overline{\eta },\overline{\mu })$ is a lifting of $(T,\eta ,\mu )$. First, we instantiate the equivalence (15) to:

17

where $[\mathsf {coalg}(B),\mathsf {Set}]^c$ is the category of countably accessible functors from $\mathsf {coalg}(B)$ to $\mathsf {Set}$.

Next, we observe there is an isomorphism as in the horizontal path below, constructed from the (unnamed) assumed isomorphisms (some are actually equalities) in the cube (drawn with double lines):

18

The functors *T* and $\overline{T}$ are both accessible by Lemma B.2. The forgetful functor $U :\mathsf {coalg}(B) \rightarrow \mathsf {Set}$ always preserves colimits, so it is accessible as well. Hence *TU* and $U\overline{T}$ are accessible. Now, by the equivalence (17), it is not difficult to show that there is a natural isomorphism $\alpha$ making the above crescent commute.

This proves that $\overline{T}$ is a lifting of *T*. It remains to show that the monad $(\overline{T}, \overline{\eta },\overline{\mu })$ is a lifting of $(T,\eta ,\mu )$. That follows from the equivalence (17) once we show that the following two diagrams commute:

Commutativity essentially follows from the lifting of $(T_c, \eta ^c,\mu ^c)$ to $(\overline{T}_c, \overline{\eta }^c, \overline{\mu }^c)$ and the two extensions, and a straightforward but tedious computation, by unfolding $\alpha \overline{I}$ as in (18). $\square$

## Footnotes

1 On $\mathsf {Set}$, the countably presentable objects are precisely the countable sets.
